# “Petal-like” size-tunable gold wrapped immunoliposome to enhance tumor deep penetration for multimodal guided two-step strategy

**DOI:** 10.1186/s12951-021-01004-1

**Published:** 2021-09-27

**Authors:** Yanan Li, Wenting Song, Yumin Hu, Yun Xia, Zhen Li, Yang Lu, Yan Shen

**Affiliations:** 1 State Key Laboratory of Natural Medicines, Center for Research Development and Evaluation of Pharmaceutical Excipients and Generic Drugs, Department of Pharmaceutics, School of Pharmacy, China Pharmaceutical University, Nanjing, 210009 People’s Republic of China; 2 School of Food Science and Pharmaceutical Engineering, Nanjing Normal University, Nanjing, 210023 People’s Republic of China; 3 Laboratory of Traditional Chinese Medicine, School of Chinese Materia Medica, Beijing University of Chinese Medicine, Beijing, 100029 China

**Keywords:** Plasmon resonance structures, Photothermal conversion, Cyclopamine, HER2, Synergistic, Gold nanoparticles

## Abstract

**Background:**

Breast cancer is the fastest-growing cancer among females and the second leading cause of female death. At present, targeted antibodies combined with hyperthermia locally in tumor has been identified as a potential combination therapy to combat tumors. But in fact, the uniformly deep distribution of photosensitizer in tumor sites is still an urgent problem, which limited the clinical application. We reported an HER2-modified thermosensitive liposome (immunoliposome)-assisted complex by reducing gold nanocluster on the surface (GTSL-CYC-HER2) to obtain a new type of bioplasma resonance structured carrier. The HER2 decoration on the surface enhanced targeting to the breast cancer tumor site and forming irregular, dense, "petal-like" shells of gold nanoclusters. Due to the good photothermal conversion ability under near-infrared light (NIR) irradiation, the thermosensitive liposome released the antitumor Chinese traditional medicine, cyclopamine, accompanied with the degradation of gold clusters into 3–5 nm nanoparticles which can accelerate renal metabolism of the gold clusters. With the help of cyclopamine to degrade the tumor associated matrix, this size-tunable gold wrapped immunoliposome was more likely to penetrate the deeper layers of the tumor, while the presence of gold nanoparticles makes GTSL-CYC-HER2 multimodal imaging feasible.

**Results:**

The prepared GTSL-CYC-HER2 had a size of 113.5 nm and displayed excellent colloidal stability, photo-thermal conversion ability and NIR-sensitive drug release. These GTSL-CYC-HER2 were taken up selectively by cancer cells in vitro and accumulated at tumour sites in vivo. As for the in vivo experiments, compared to the other groups, under near-infrared laser irradiation, the temperature of GTSL-CYC-HER2 rises rapidly to the phase transition temperature, and released the cyclopamine locally in the tumor. Then, the released cyclopamine destroyed the stroma of the tumor tissue while killing the tumor cells, which in turn increased the penetration of the liposomes in deep tumor tissues. Moreover, the GTSL-CYC-HER2 enhanced the performance of multimodal computed tomography (CT) and photothermal (PT) imaging and enabled chemo-thermal combination therapy.

**Conclusions:**

This optically controlled biodegradable plasmonic resonance structures not only improves the safety of the inorganic carrier application in vivo, but also greatly improves the anti-tumor efficiency through the visibility of in vivo CT and PT imaging, as well as chemotherapy combined with hyperthermia, and provides a synergistic treatment strategy that can broaden the conventional treatment alone.

**Graphic Abstract:**

**Figure:**
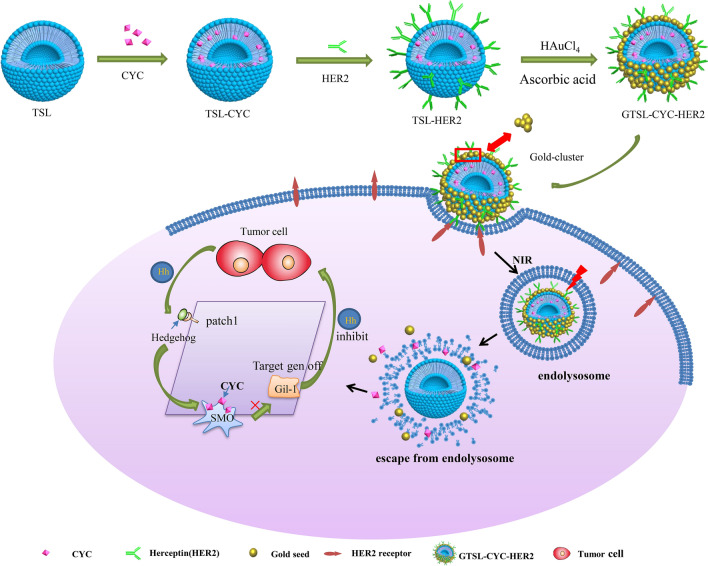

**Supplementary Information:**

The online version contains supplementary material available at 10.1186/s12951-021-01004-1.

## Introduction

Clinically, more than 30% of breast cancer patients overexpressed HER2 protein, which always indicated poor differentiation, rapid proliferation, high distant metastasis rate and poor prognosis [1]. Therefore, HER2 antibody-based monoclonal antibody has been widely used in improving the therapeutic effect of HER2-positive breast cancer patients, gaining great clinical benefit [2]. However, to achieve longer tumor remission period, the clinical regimen also introduced the combination of HER2 antibody monoclonal antibody with chemotherapeutic or photothermal drugs, such as docetaxel, paclitaxel, photosensitizer and other anthraquinone anticancer drugs. Moreover, Her2 antibody-mediated molecular targeted therapy by linking HER2 antibodies with biologically active molecules to form antibody–drug conjugates has also been rapidly developed, which improve the therapeutic index and minimize off-target side effects in normal tissues. Generally, enabling the HER2 antibody to be used in multi-modality therapy and targeted delivery still possesses huge potential.

At present, targeted antibodies combined with hyperthermia locally in tumor has been identified as a potential combination therapy to combat tumors. Recently, gold nanostructures have attracted great interest in the hyperthermia therapy due to their properties such as local surface plasmon resonance (LSPR), photo-thermal transformation efficiency [3–5]. But in fact, the uniformly deep distribution of photosensitizer in tumor sites is still an urgent problem, which limited the clinical application [6]. As we know, after the drug or even nanoparticles reach the tumor site, it stays more in the tumor matrix near the tumor blood vessel, and is difficult to reach the deep area of the tumor to realize the uniform distribution of photosensitizer and total collapse of tumor. The dense extracellular matrix (ECM) in solid tumors is one of the reasons for increasing the solid pressure inside the tumor [7], which hinder the penetration of carrier in tumor tissues. For example, the dense vascular network and the reduction of blood flow limit the convection of nanoparticles (NPs), and also extend the diffusion path of NPs from blood vessels to tumor cells. The thickness of fibronectin and collagen in ECM limits the diffusion of NPs [8]. Therefore, many studies are devoted to down-regulating the expression of tumor ECM or selectively degrading the formed ECM [9].

Studies have confirmed [10] that the Hh signal transduction pathway is activated in breast cancer and plays an important role in the occurrence and development of breast cancer. Cyclopamine (CYC) is a traditional Chinese medicine, heterosteroidal alkaloids isolated from plants of the genus Veratrum. It can inhibit the Hh signaling pathway by acting on SMO receptors [7, 11], which in turn can destroy the synthesis of tumor ECM. Jiang et al. [12]. showed that the expression of fibronectin in tumor tissues was significantly after CYC treatment. They found significantly improved tumor perfusion and reduced interstitial fluid pressure due to the destruction of the synthesis of tumor ECM. Hence, in addition to act as an anti-tumor Chinese drug, cyclopamine could also help the transport of other drug in tumor tissue and promotes the accumulation and distribution. However, cyclopamine was limited in its clinical application due to poor solubility. Drug delivery vectors, such as liposome, micelle or nanoparticles, are needed to improve the bioavailability of cyclopamine [13].

**Fig. 1:**
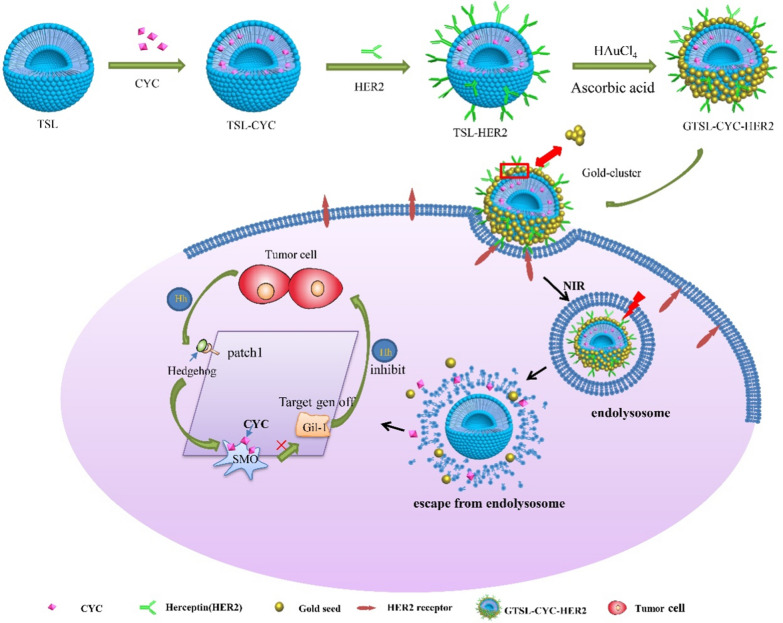
Illustration of dual-targeting GTSL-CYC-HER2 and its intracellular trafficking pathway in the tumor cells

In our study, two strategies are introduced to achieve deep penetration and the synergistically enhanced antitumor effect for combination therapy. The first step is to construct a plasmon resonance nano-structure that has size-tunable degradability. We firstly grafted the thermosensitive liposome surface with Her2 antibody, which is further decorated with a layer of gold nanoclusters, forming a biodegradable plasmon resonance structure. Under NIR irradiation, the gold nanoclustered liposome can selectively release gold nanoparticle to realize the size and morphology transformation in the tumor microenvironment through photothermal effect. This size-tunable ability combine the following advantages, including the tumor targeting of large particles(> 100 nm) and tumor diffusion of small particles (< 30 nm) for in-depth internal treatment of tumors. Secondly, the chemotherapeutic drug, cyclopamine encapsulated in liposomes could degrade ECM to further improve the diffusion of gold nanoparticles. Besides, liposome also improves the druggability of cyclopamine. Based on above system (Fig. 1), gold nanocluster-coated thermosensitive immunoliposome drug delivery system (GTSL-CYC-HER2), it can completely kill the tumor from the outer tumor tissue to deep cores by integrating hyperthermia, chemotherapy and degrading matrix therapy.

**Fig. 2:**
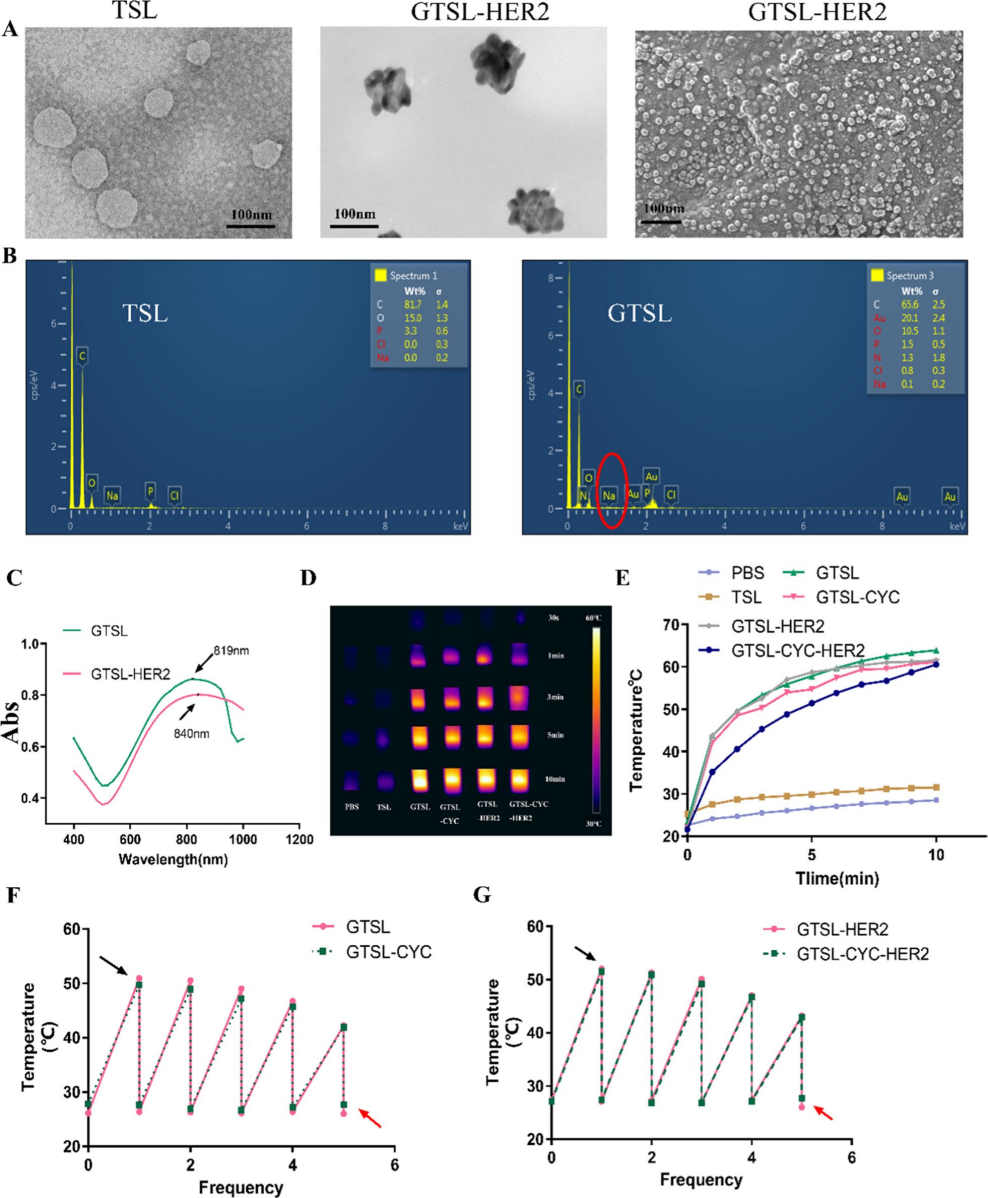
**A **Representative TEM and SEM images of different nanoparticles. (Scale bar: 100 nm). **B** The EDS spectrum of TSL and GTSL. **C** The UV − vis absorption spectra of GTSL and GTSL-HER2. **D **The photothermal image of GTSL,GTSL-CYC,GTSL-HER2,GTSL-CYC-HER2. **E** Temperature change of PBS, TSL, GTSL, GTSL-CYC, GTSL-HER2 and GTSL-CYC-HER2. Temperature increments of GTSL, GTSL-CYC (**F**) and GTSL-HER2, GTSL-CYC-HER2 (**G**) after 5 repeated irradiations (black arrow indicates temperature before illumination and red arrow indicates temperature after illumination)

## Results and discussion

### The grafting ratio of HER2 antibody to thermosensitive liposome

Firstly, the HER2 was investigated to be grafted onto the surface of TSL via amide bond or disulfide bond, respectively. As shown in Table.1, the grafting efficiency by amine bond (86.53%) was much higher than that by disulfide bond (3.33%). It is speculated that both the molecular weight and steric hindrance in space of HER2 antibody is large, which hinder the rapid reaction by disulfide bond. Meanwhile, DSPE-PEG2000-SH is more likely to self-cross-link, resulting in low grafting efficiency with HER2 antibody. Therefore, 1:100 (DSPE-PEG2000-COOH: HER2) was the optimal ratio for amine bond connection, which is used for further experiments. To further confirm the existence and integrity of amine-bound HER2 antibody, the SDS-PAGE electrophoresis was carried out. The results showed that the free HER2 antibody existed two bands at 50kD and 25kD, representing heavy chain CH1 and light chain VH. Then, similar bonds at 50kD and 25kD also appeared in the DSPE-PEG-HER2, TSL-HER2 and TSL-CYC-HER2 sample, indicating that hydrophobic DSPE domain of DSPE-PEG-HER2 had been spontaneously incorporated into the liposome lipid bilaterals (Additional file 1: Figure S1).

**Table 1:** Ligation efficiency of antibodies in different immunoliposomes

Connection method	molar ratio	Added Protein content (μg/mL)	Measured Protein content (μg/mL)	Connection efficiency (%)
Amide bond	1:25	3.2	0.083	2.60 ± 2.10
	1:50	1.6	0.343	21.43 ± 3.44
	1: 100	0.8	0.692	86.53 ± 6.78
	1: 200	0.4	0.316	78.90 ± 6.15
	1: 400	0.2	0.150	74.84 ± 7.04
Disulfide bond	1:25	3.2	0.0154	0.48 ± 0.22
	1:50	1.6	0.015	0.28 ± 0.13
	1: 100	0.8	0.004	1.38 ± 1.01
	1: 200	0.4	0.011	1.94 ± 0.89
	1: 400	0.2	0.008	3.33 ± 2.33

### The characteristics of gold nanocluster-coated thermosensitive immunoliposome drug delivery system (GTSL-CYC-HER2)

As shown in Fig. 2A, compared with TSL, the particle size of HER-GTSL did not change significantly after in situ reduction by chlorauric acid and modification by HER2, due to the electrostatic effect of gold nanoclusters which made the liposome tight. However, after mixing with HAuCl_4_, the reducing agent could reduce Au^3+^ ions to neutral gold ions, which were wrapped on the surface of liposome and shield the negative charge of liposome. Attributed to wrapping of gold nanoclusters (GN), the zeta potential of liposomes changed from − 25.87 ± 6.15 mV to almost electrically neutral. Hence, the gold wrapped liposome presented electric neutrality. Moreover, the TEM images showed that primary TSL exhibited uniformly smooth surface, while GTSL-CYC-HER2 presented a special "petal-like" structure, which could be explained by the continuous aggregation of gold nanoclusters on the surface of liposomes, forming irregular petal-like shells of gold nanoclusters on the surface of liposomes (Fig. 2A). We found the coating level of GN on TSL was controlled by the concentrations of the GN precursors (Additional file 1: Figure S2). When 24 μl of HAuCl_4_ and 36 μl of ascorbic acid was mixed with 1 ml TSL, the special thickness of GN could turn the UV absorption wavelength from 600 to 800 nm, which is suitable for the deep penetration of light [14] (Additional file 1: Table S1).

To further determine the binding of GN on TSL, energy dispersive X-ray spectroscopy was used to detect the element peak of Au. Different from the TSL, the results indicated that an obvious Au peak appeared in the EDS spectrum of GTSL (Fig. 2B). It is noted the position of Au and P in phospholipids was similar, indicating the co-localization of phospholipids and gold phosphorus. Therefore, the gold nanoclusters were successfully modified on the surface of liposomes. Furthermore, UV − vis absorption spectra demonstrated that GTSL and GTSL-HER2 displayed distinct absorption peak at 819 nm and 840 nm, respectively (Fig. 2C), which confirmed the excellent localized surface plasmon resonance (LSPR). Thus, the photo-electronic converting property of the GTSL-CYC-HER2 was explored. As shown in Fig. 2D and E, the extraordinary photothermal performance of GTSL, GTSL-CYC, GTSL-HER2, GTSL-CYC-HER2 was confirmed as the solution temperature rose rapidly from 22 to 40.5 ℃, 39.2、40.2 and 38.9 ℃ within 10 min, respectively, while a barely photothermal effect was observed in PBS and TSL groups. Moreover, the loading of CYC inside GTSL cause no effect on the LSPR properties of GN. Besides, the photothermal effect of GTST and GTSL-CYC keep stable in a 5-cycle irradiation study, only witnessing a slight decay in the fifth photothermal conversion compared with the first irradiation (Fig. 2F, G). This observation indicated that the TSL could greatly conserve the photothermal conversion efficiency of GN. Further, with the continuous repeat of irradiation times, the GN on the surface of TSL will be broken into small gold nanoparticles, which reduced the photothermal conversion ability. However, the small particles could be easily cleared by kidney metabolism with less accumulated toxicity in vivo. Finally, after drawing the linear relation between the cooling time and –lnθ (Additional file 1: Figure S3), the photothermal conversion efficiency of GTSL and GTSL-CYC-HER2 were calculated by the formula as 30.14% and 37.96% respectively (Additional file 1: Table S2). This result was higher than the reported photothermal materials [15], including the gold nanorods (~ 21%), gold nanoparticles (~ 11%), MoS2 nanoplates (~ 27.6%) and Bi nanoparticles (~ 30%). Considering the TSL encapsulated in the GN, the GTSL can be regarded as a hollow gold nanostructure with a larger kernel size. Hence, the photothermal conversion efficiency was higher under NIR irradiation as the specific surface area is much larger than that of other photothermal materials. As reported, the Au nanoshells could be served as a strong NIR absorber and thermal transducer, due the larger optical absorption cross-section^16^. Shanmugam et al. evaluated the photothermal destruction of rod-in-shell Au to cancer cells, which revealed that rod-in-shell particles exhibit a more effective anticancer efficacy in the laser ablation of solid tumors compared to Au NRs [17]. Furthermore, for the comparison of Au nanorods and Au nanoshell, Cheng et al. found that the photothermal effect was more effective in Au nanoshells than Au nanorods [18], which further confirmed the superior photothermal effect of GTSL-HER2.

### The photothermal responsive release behavior and degradation of GTSL-CYC-HER2

**Fig. 3:**
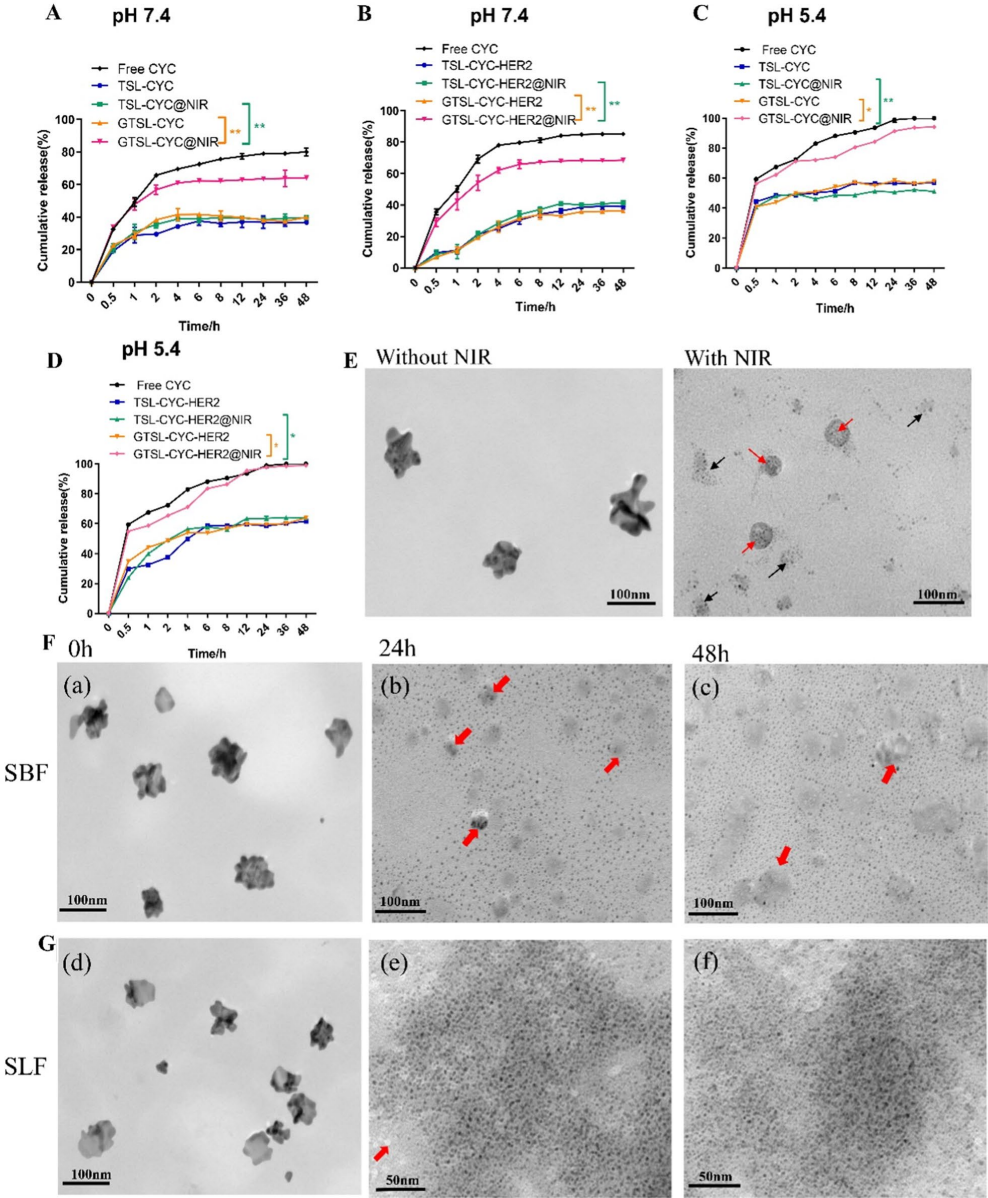
In vitro release of free CYC, TSL-CYC, GTSL-CYC and free CYC, TSL-CYC-HER2 and GTSL-CYC-HER2 with or without NIR in pH 7.4 (**A**–**B**) and in pH 5.4 (**C**–**D**) (*p < 0.05, **p < 0.01). **E** Degradation behaviors of GTSL-CYC-HER2 irritated without or with NIR. (red arrows indicate gold nanoclusters, black arrows indicate free gold nanoseeds) **F** Degradation behaviors of GTSL -CYC-HER2 in SBF (a–c) and SLF (d–f) observed by TEM at 0, 24, 48 h. (Red arrows indicate liposome fragments) (a–d: Scale bar = 100 nm. e–f: Scale bar = 50 nm)

We next examined the photothermal responsive release behavior of GTSL-CYC-HER2 triggered by manually controlled NIR irradiation in simulated different humoral conditions in vitro. Firstly, all the liposome preparations showed a sustained release in pH 7.4 compared with free CYC (4 h, 69.61%) (Fig. 3A, B). Among that, TSL-CYC, TSL-CYC@NIR, TSL-CYC-HER2, TSL-CYC-HER2@NIR, GTSL-CYC and GTSL-CYC-HER2 achieved 36.75, 39.81, 39.04, 41.57, 39.93, and 36.33% release within 48 h, while GTSL-CYC@NIR and GTSL-CYC-HER2@NIR reached 64.20 and 68.44% in 48 h, suggesting that the manually controlled NIR irradiation could accelerate the release of encapsulated CYC in the TSL (Fig. 3A, B). It is noted the cumulative release of CYC in the first 2 h appeared a burst release with 56.86% and 54.18% in GTSL-CYC@NIR and GTSL-CYC-HER2@NIR groups. The above results indicated that GTSL-CYC-HER2 could effectively convert light energy into heat and the temperature reached phase transition temperature under 808 nm NIR irradiation, which promoted the release of CYC from TSL. Next, to observe the release behavior in the lysosome, the PBS at pH5.4 was chosen as the release medium. As shown in Fig. 3C, D, the release profile indicated that there was 57.02%, 51.09%, 61.58% and 63.99% of CYC liberated from TSL-CYC, TSL-CYC@NIR, TSL-CYC-HER2 and TSL-CYC-HER2@NIR groups at 48 h, suggesting a faster release effect in lysosome. The cumulative release in 48 h was 94.15% for GTSL-CYC and 98.85% for GTSL-CYC-HER2 in lysosome under the laser irradiation. Attributed to the acidic lysosomal environment, the stability of TSL was further reduced and CYC almost completely released.

**Fig. 4:**
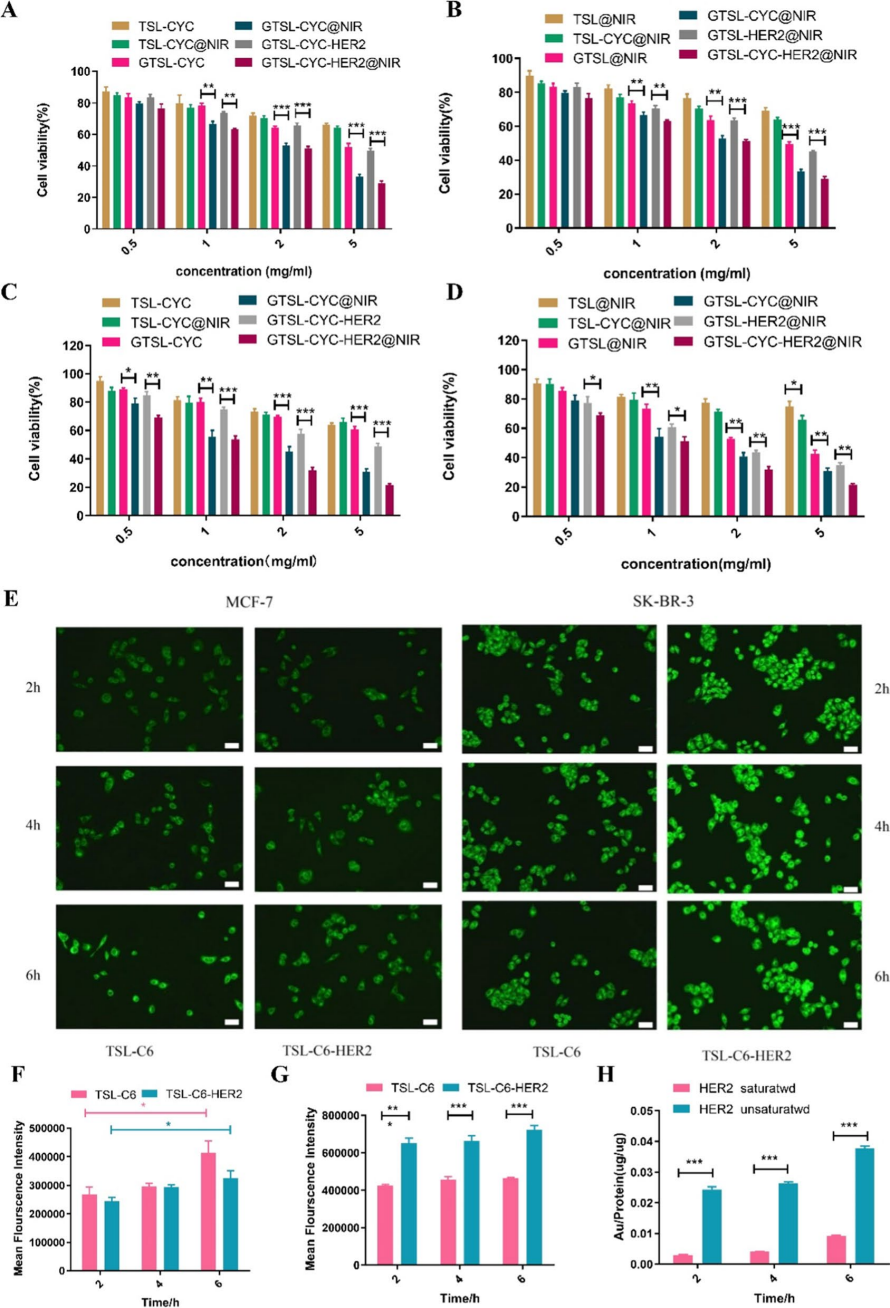
MCF-7 Cell viability of the synergy group compared with the chemotherapy groups (**A**) and hyperthermia groups (**B**). (**p < 0.01, ***p < 0.001).SK-BR-3 Cell viability of the synergy group compared with the chemotherapy groups (**C**) and hyperthermia groups (**D**). (*p < 0.05, **p < 0.01, ***p < 0.001) (E) MCF-7 cell uptake of TSL-C6 and TSL-C6-HER2 and SK-BR-3 cell uptake of TSL-C6 and TSL-C6-HER2 (Scale bar = 50 μm). Mean fluorescence intensity of TSL-C6 and TSL-C6-HER2 in SK-BR-3 cells (**F**) and MCF-7 cells (**G**) (*p < 0.05, **p < 0.01, ***p < 0.001). **H** Quantitative comparation of GTS-CYC-HER2@NIR uptaken into SK-BR-3 cells with or without HER2 antibody blocking (***P < 0.001)

**Fig. 5:**
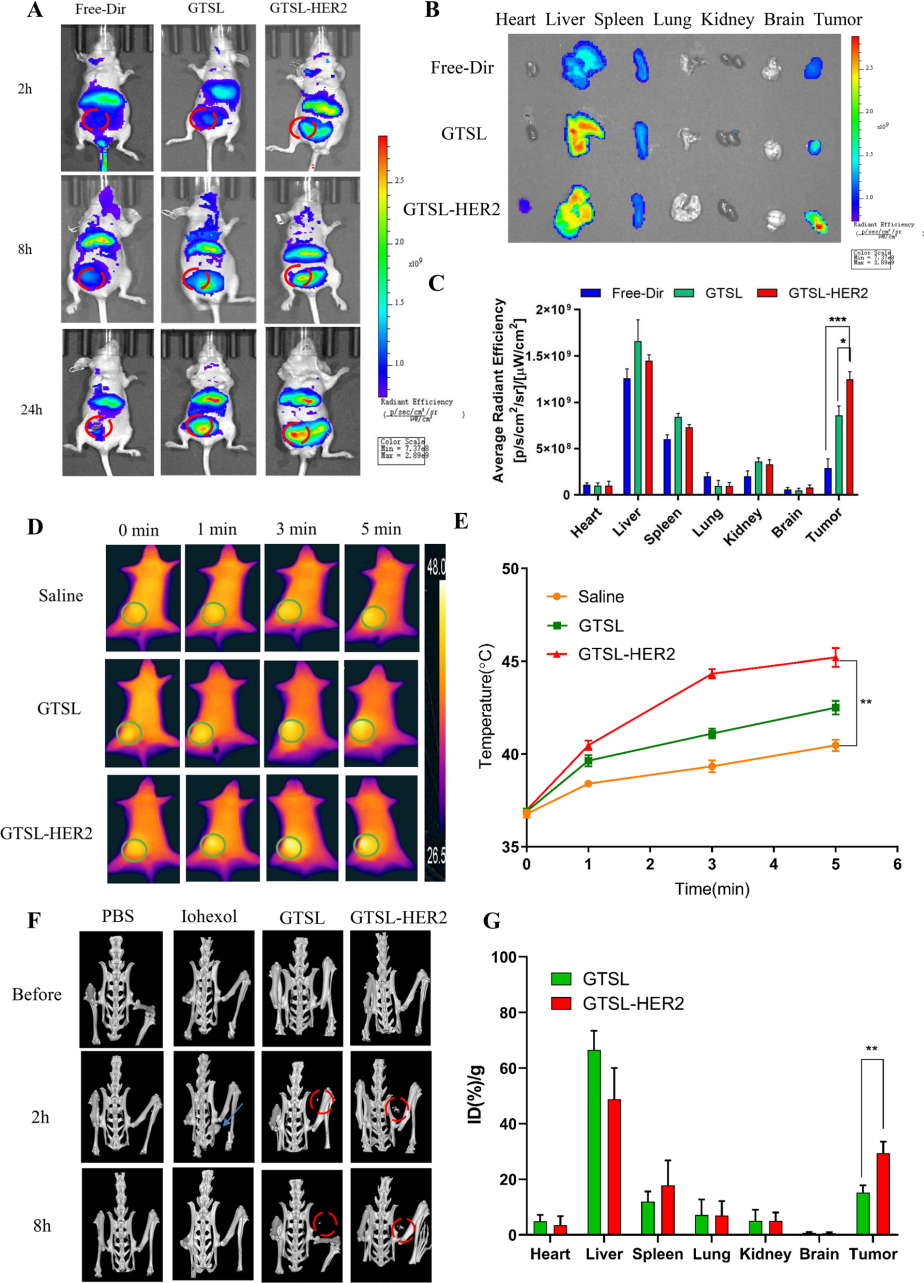
**A** The in vivo imaging of SK-BR-3 tumor bearing mice after intravenous injection of free Dir, Dir loaded GTSL, and GTSL-HER2. (Red circles represent the tumor site). **B** The images of ex vivo organ of mice at 24 h after injection free Dir, Dir loaded GTSL, and GTSL-HER2. **C** The average fluorescence intensity of ex vivo organs and tumors of mice at 24 h after injection of different groups (n = 3, mean ± SD). (*p < 0.05, ***p < 0.001). **D** In vivo photothermal imaging with a NIR laser at 0, 1, 3 and 5 min of SK-BR-3 tumor-bearing mice 24 h after intravenous injection of saline, GTSL and GTSL-HER2. (Green circles represent the tumor site). **E** Temperature changes of the SK-BR-3 tumor-bearing mice during NIR irradiation at different points of different groups (n = 3, mean ± SD). (**p < 0.01). **F** The in vivo CT imaging of SK-BR-3 tumor bearing mice after intravenous injection of PBS, Iohexol, GTSL, and GTSL-HER2. (Red circles represent the tumor site). **G** The average Au amount of ex vivo organs and tumors of mice at 8 h After injection of different groups (n = 3, mean ± SD). (**p < 0.01)

**Fig. 6:**
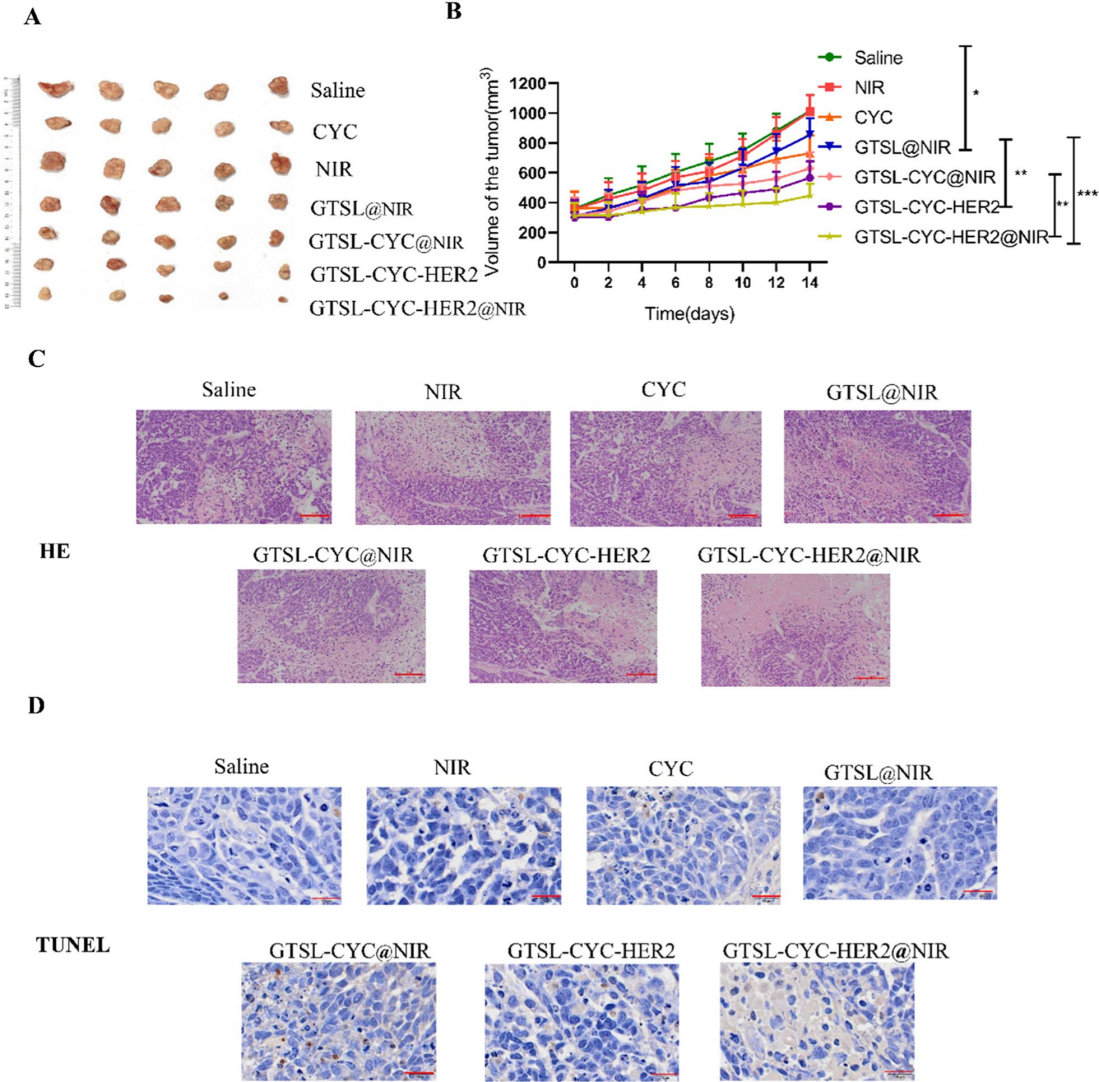
**A**The tumor images of different treatment groups at 14 day treatment. **B** Tumor volume changes in different treatment groups within 14 days. groups (n = 5, mean ± SD). (*p < 0.05, **p < 0.01, ***p < 0.001). **C** Representative H&E staining images of tumor tissues after different treatments (Scale bar = 100 μm).** D** TUNEL images of tumor tissues after different treatments (Scale bar = 100 μm)

**Fig. 7:**
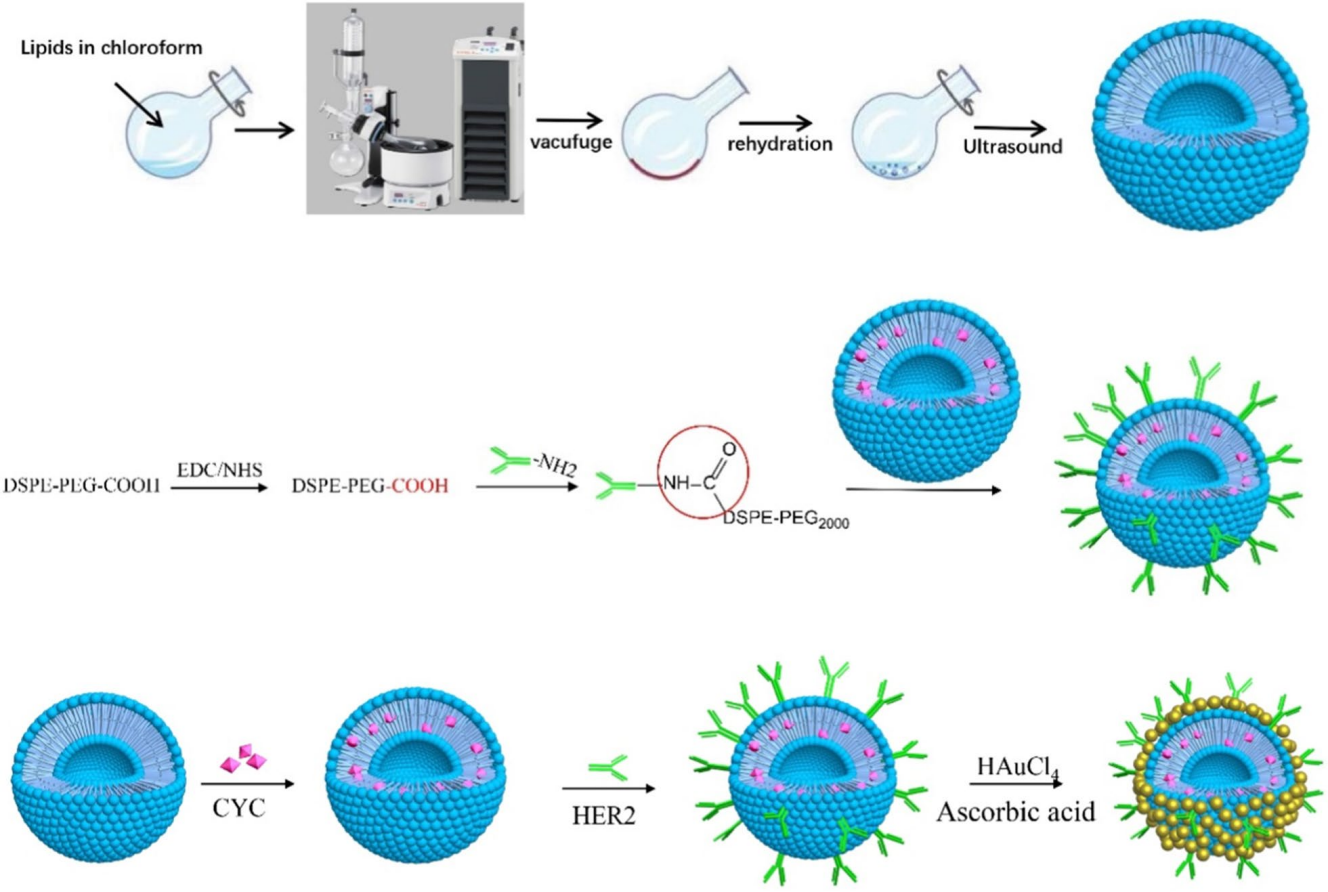
Preparation of drug-loaded thermosensitive immunoliposomes coated with gold nanoclusters

To further observe the degradation of GTSL-CYC-HER2, the morphology of the particles under different conditions was recorded. Clearly, the GTSL-CYC-HER2 presented "petal-like" with a clean background without laser irradiation (Fig. 3E). However, after irradiation, the "petal-like" structure turned into spherical shape. It is speculated when the temperature rose rapidly to the phase transition temperature accompanied with unstable lipid membrane, the gold nanoclusters on the surface gradually turned into free small gold nanoparticles of 5-8 nm. Furthermore, the degradation experiment was also performed in simulated humoral conditions (blood solution and lysosome solution). From Fig. 3F, we can see that GTSL-CYC-HER2 degraded gradually in neutral simulated blood fluids (SBF). From 0 to 24 h, the GN mostly separated from the TSL-HER2 while the liposome still kept the spherical structure. As we know, gold nanoclusters could be degraded into 5–8 nm gold particles in situ on the surface of liposomes if the liposomes were collapsed. After 24 h incubation in SBF, the liposomes will also be unstable and degrade to some extent, leading to the dissociation of some gold nanoclusters. But from 24 to 48 h, all the GN completely liberated from the TSL and the liposome mostly broke apart to release CYC. However, the GTSL-CYC-HER2 began to appear as a unstable precipitate within 24 h in simulated lysozyme fluids (SLF) becoming the free GNs and broken liposome fragments (Fig. 3G). The degradation rate was sharply faster than that in SBF solution due to the enhanced β-ester hydrolysis of phospholipids in the acid environment, indicating the good biocompatibility. Therefore, the gold nanoclusters not only prolonged their biodegradation rate by stabilizing the liposome core, but also were metabolized to 5–8 nm particles to be cleared by kidneys. This controllable and biodegradable complex provided a great platform for synergistic treatment of photothermal therapy and chemotherapy.

### The in vitro cellular evaluation of GTSL-CYC-HER2 at cellular level

We first evaluated the expression of HER2 protein among four different cancer cells by western blot and immumofluorescence methods, including MCF-7, MDA-MB-453, BT474 and SK-BR-3 cells. According the expression content (Additional file 1: Table S3; Figure S4), MCF-7 was chosen as the low-expression cell and SK-BR-3 as the high-expression cell of HER-2 for further cellular evaluation experiments. In addition, the single irradiation with power of 1 or 3 w/cm^2^ had no significant effect on the viability of cells while the power of 5 w/cm^2^ caused 10–15% mortality (Additional file 1: Figure S5). Considering the photothermal effect and safety, 3 w/cm^2^ was chosen as the optimal irradiation power for further experiments.

The cytotoxicity was tested by 3-(4,5)-dimethylthiahiazo (-z-y1)-3,5-di- phenytetrazoliumromide (MTT) assay to evaluate the synergistic therapeutic effect on MCF-7 and SK-BR-3 cells. Firstly, in MCF-7 cells, there was almost no significant difference between the synergic treatment group and single therapy groups at the low concentration of 0.5 mg/ml (Fig. 4A, B). This finding was ascribed to the low content of CYC and GNs in the TSL which as unable to produce effective chemotherapeutic response and photothermal conversion ability. With the increase of liposome concentration, both the CYC inside TSL and GNs on the TSL concentration increased, the advantages of synergistic therapy began to emerge. The inhibitory effects of GTSL-CYC-HER2@NIR on MCF-7 cells were significantly higher than those of the single chemotherapy group and the single photothermal group (**p < 0.01).When the liposome concentration reached 5 mg/mL, the average inhibition percent of GTSL-CYC-HER2@NIR were 68.65% and 70.67%, respectively, which were much higher than the average inhibition percent of GTSL-CYC (49.67%) and GTSL-CYC-HER2 (51.05%), respectively(***p < 0.001). It was also much higher than that for GTSL@NIR (49.59%) and GTSL-HER2@NIR (51.69%)(***p < 0.001) (Fig. 4A, B). All the above results indicated that the synergistic photothermal and chemotherapy could significantly inhibit the proliferation of MCF-7 cells. Next, compusyn software was introduced to calculate the synergistic treatment index, shown in Additional file 1: Table S4. Generally, the synergistic index CI is basically less than 1, which confirmed the synergistic “1 + 1 > 2” effect of chemotherapy combined with hyperthermia for GTSL-CYC-HER2, instead of a simple superposition effect. With liposome concentration increasing, CI further declined, demonstrating an enhanced synergy (Additional file 1: Figure S6). Besides, similar results were obtained with SK-BR-3 cells (Fig. 4C, D and Additional file 1: Figure S7). However, it is noted that HER2-decorated GTSL-CYC-HER2@NIR exhibited superior higher killing ability than GTSL-CYC@NIR in SK-BR-3 cells, which is attributed to the high expression of HER2 and enhanced targeting (Additional file 1: Table S5).

### The targeting evaluation of GTSL-CYC-HER2 in vitro

Next, we further investigated the cellular uptake behavior of GTSL-CYC-HER2@NIR determined by fluorescence inverted microscope and flow cytometry. As shown in Fig. 4E, during 6 h incubation, the increasingly accumulated fluorescence was detected in MCF-7 cells, revealing the time-dependent uptake. At the same time point, the fluorescence of TSL-C6-HER2 is similar with that of TSL-C6, suggesting the HER2 coating could not enhance the uptake by MCF-7 cells. However, the significant difference of fluorescence appeared between the TSL-C6 and TSL-C6-HER2 groups in SK-BR-3 cells (Fig. 4E–G). We further confirmed the HER2-mediated active uptake by receptor saturation inhibition assay. After saturation by excessive HER2 solution, the uptake of GTSL-CYC-HER2 dramatically reduced with 6 h in SK-BR-3 cells (Fig. 5H). All the results demonstrated that GTSL-CYC-HER2 could be effectively transferred into HER2-expressing tumor cells.

Then, to test the uptake ability of gold element by tumor cells, the Au/protein at different point was detected via ICP-MS. In MCF-7 cells, the laser irradiation was able to improve the uptake of Au by MCF-7 cells (Additional file 1: Figure S8A). This phenomenon may be due to that heat generated by laser excitation can promote the fluidity of cell membrane and thus improve the uptake of GNs [19, 20]. Furthermore, the free GNs separated from TSL were more favorable for endocytosis process with smaller size [21, 22]. The same trend was found in the uptake assay in SK-BR-3 cells. Different from that in MCF-7 cells, a significantly increased uptake rate of Au was observed in GTSL-CYC-HER2 (***p < 0.001) (Additional file 1: Figure S8B, C), compared with that in GTSL-CYC, which further indicated the active targeting to HER2-positive cells.

Finally, we further established a tumor-sphere model to investigate whether the adjustment of gold nanoparticle size induced by NIR had a positive effect on tumor penetration. As shown in Additional file 1: Figure S9, the results showed that the penetration depth of GTSL-F@NIR increased from 40 to 80 μm compared with that of the GTSL-F, indicating that the particle size reduction induced by irradiation was beneficial to the penetration into the tumor sphere.

### In vivo targeting and antitumor efficacy

It is reported that HER-2-decorated liposome can target to tumor sites owing to the passive EPR effect and active receptor-mediated endocytosis effect. The targeting ability of GTSL-CYC-HER2 was firstly verified by in vivo imaging system by labeling TSL with Dir. As presented in Fig. 5A–C and Additional file 1: Figure S10, after intravenously injecting Dir@GTSL-HER2, the increased accumulated Dir fluorescence was detected in tumor area within 24 h, while the fluorescence of free Dir group suffered from a rapid decline. Fluorescence images of all organs at 24 h further showed the fluorescence of the GTSL-HER2 group was 1.5 times that of the GTSL group and five times that of the free Dir group, suggesting the longer retention time of GTSL-HER2 in the tumor site. These results clarified that GTSL-HER2 could actively target to tumor area and be delayed in tumor. Besides, it is noted the liposome preparations also tended to be accumulated in the liver and kidneys, compared with free Dir group. This phenomenon may be due to the enrichment of blood vessels in these metabolic organs, which is consistent with the reported results [23]. Moreover, according to the reports, glomerulus could filter nanoparticles with a hydrodynamic diameter of smaller than 6 nm size [24]. For instance, insulin with a hydrodynamic size of approximately 3 nm can be totally excreted through the urinary system with 100% filtering performance. However, the neutral-charge surface modification could also help proper renal excretion of NPs with a hydrodynamic size of approximately 6–8 nm [24], due to sensitivity of the glomerular endothelial wall to the neutral surface charge of NPs [25]. In our study, after irradiation, the gold nanoclusters on the surface gradually turned into free small gold nanoparticles of 5–8 nm. Moreover, the surface of gold nanoparticles is electrically neutral which further demonstrated that the gold NPs could be cleared by kidneys. The Figure of the in vivo distribution at 96 h were shown in Additional file 1: Figure S11. As we can see that there's no residue of the preparations in the kidneys. In our study, we also detect the Au content in kidneys at 8 h and 168 h after injection. The content of the Au at 8 h was 4.98 ID(%)/g and the levels of Au in the kidneys were undetectable at 168 h after injection of GTSL-HER2.

Based on the superior targeting ability, the localized photothermal property in tumor was determined by near infrared photothermal imaging technology after intravenously injection of different preparations for 24 h. Compared to the unobvious temperature increase in the saline group and GTSL group, the tumor temperature in mice treated with GTSL-HER2 quickly reached up to 45℃ with 5 min of laser irradiation (3 W/cm^2^), indicating the superior photothermal conversion ability(**p < 0.01) (Fig. 5D, E). In addition, the photothermal imaging caused by NIR can also monitor the treatment process in real time, which provides new platform for the evaluation of therapeutic efficacy.

Compared with iodine, gold has a higher atomic number, indicating strong X-ray attenuation ability and great potential in CT imaging [26]. Herein, we also established in situ breast cancer model to verify the tumor-targeted CT imaging ability. After intravenously injection of different preparations, Fig. 5F and G showed that the brightness of the tumor site did not change significantly with the extension of time in iodothyol solution group. However, the obvious brightness appeared in the bladder after 2 h and then disappeared fastly, indicating that iodothyol was easy to be metabolized and cleared in vivo. Excitingly, the tumor site in mice treated with GTSL and GTSL-HER2 evidenced an enhanced contrast effect at both 2 and 8 h. And the intensity of GTSL-HER2 was significantly higher than that of GTSL, suggesting the GTSL-HER2 possessing better targeted contrast effect. Next, we determined the content of Au in the organs and found that although Au element was mostly accumulated in the liver and spleen, the Au content of GTSL-HER2 was obviously more than that in other groups, clarifying the targeted CT imaging potential.

Above results all demonstrated the good targeting ability and excellent photothermal performance. Then, we evaluated the antitumor effect in the in situ breast cancer-bearing mice. Figure 6A and B showed that NIR group exhibited negligible tumor suppression after treatment for 14 days. In addition, the single therapy, such as GTSL@NIR or CYC group, only showed modest antitumor effect, which revealed the limitation of single therapy. However, compared to GTSL-CYC, the tumor in GTSL-CYC-HER2 group was further inhibited, which could be attributed to the enhanced active targeting with HER2 decoration. Moreover, significant tumor ablation was observed in the combined treatment group, especially GTSL-CYC-HER2@NIR group. This amplified antitumor effect was due to the following reasons. Firstly, under near-infrared laser irradiation, the temperature of GTSL-CYC-HER2 rises rapidly to the phase transition temperature, and released the cyclopamine locally in the tumor. Then, the released cyclopamine destroyed the stroma of the tumor tissue while killing the tumor cells, which in turn increased the penetration of the liposomes in deep tumor tissues. This strategy accelerated the process of tumor collapse just like peeling the onion layer by layer. Moreover, the weight of mice in all groups kept stable without significant difference, and all organs showed no obvious injury, suggesting potentially good biosafety during therapy in vivo (Additional file 1: Figures S12, S13). Finally, Hematoxylin and eosin (H&E) staining of tumor tissue showed much more severe tumor cell damage in GTSL-CYC-HER2@NIR group, because of the obvious cell shrinkage and nuclear condensation (Fig. 6C). Terminal deoxynucleotidyl transferase dUTP nick end labeling (TUNEL) assay further indicated the most obvious tumor cell apoptosis of tumor cells in GTSL-CYC-HER2@NIR group (Fig. 6D). These results clearly proved the enhanced synergistic therapeutic effect by integrating the photothermal therapy, chemotherapy and disruption of tumor stroma.

## Conclusion

In summary, a gold nanocluster-coated thermosensitive immunoliposome drug delivery system by integrating the adjustment of particle size and tumor stromal collapse strategies was developed for deep and thorough tumor ablation. In our study, HER2 modification could enhance the active targeting to tumor to a great extent. Different from the photothermal property of single AuNPs, the gold nanocluster on the surface of TSL exhibited superior photothermal converting ability because of the large cavity structure. Subsequently, photothermal effect triggered the decreasement of particle size and release of cyclopamine which further enhance the penetration of this preparations, achieving a synergistic “1 + 1 > 2” effect. Ultimately, the cell and animal experiments convincingly illustrated that the GTSL-CYC-HER2@NIR had promising antitumor effect and lower side effects. The GTSL-CYC-HER2 presented here provided a promising delivery platform that realizes the controllable combination of different therapies.

## Materials and experiment methods

### Materials

Cyclopamine was purchased from Aladdin Biochemical Technology Co., LTD. DPPC, DSPE-PEG2000, DSPE-PEG2000-COOH, MPPC, HSPC and cholesterol were purchased from Shanghai RVT Pharmaceutical Technology Co., LTD. EDC and NHS were obtained from Nanjing Chemical Reagent Co., LTD. And FITC-PEG2000-SH was purchased from ponsure Co.

### The synthesis and characterization of Her2-lipid

To optimize connection efficiency of phospholipids and antibodies, two reaction strategies (amide bonds [27]and disulfide bonds [28]) were carried out referring to reports [29]. In the experiment, the molar ratios of DSPE-PEG2000-COOH and DSPE-PEG2000-SH to HER2 were 1:25, 1:50, 1:10, 1:200, 1:400 respectively. After reaction, the DSPE-PEG2000-HER2 was purified by column chromatography. The connection rate was calculated by the following formula. Besides, free HER2, DSPE-PEG-HER2 was examined by SDS-PAGE to confirm its molecular weight.

$$ {\text{Connection rate}}\left( {\text{\% }} \right) = \frac{{C_{1} }}{{C_{0} }} \times 100\% $$

### Preparation of gold nanocluster-coated and cyclopamine-loaded thermosensitive immunoliposome(GTSL-CYC-HER2)

According to the following figure, the thermosensitive liposome (TSL) and CYC-loaded TSL (TSL-CYC) were prepared by thin film dispersion method. Then, the DSPE-PEG2000-HER2 was added into above liposome solution to incubate for about 18 h at 4℃ (DSPE-PEG2000 to DSPE-PEG2000-HER2 = 1:1). Finally, the HER2 modified liposome formed after removing the free HER2 antibody with CL-4B agarose gel column.

Next, 24 μl HAUCL_4_ (100 nM) was added into TSL-HER2 solution and mixed uniformly. Then the reducing agent ascorbic acid (36 μl, 500 nM) was added into above solution and shake gently until the color of solution changes from transparent white to dark blue or dark green, suggesting that GTSL, GTSL-HER2 and GTSL-CYC-HER2 were formed (Fig. 7).

### Characteristics of GTSL-CYC-HER2

#### Size and zeta potential detection

The prepared TSL-HER2 and GTSL-CYC-HER2 were diluted to a concentration of 1 mg/mL, and then the particle size and zeta potential were determined by laser particle size analyzer (Brookhaven Instruments, USA).

#### TEM imaging

Drop the TSL-CYC and GTSL-CYC-HER2 solution on the copper net covered with carbon film for 30 s, respectively. The absorb the excess liquid with filter paper and dry it for about 3 min under the light. The morphology of the preparations was observed by transmission electron microscope and scanning electron microscope.

#### Energy dispersive X-ray spectroscopy

Add 3% sucrose as a freeze-dried protective agent to an appropriate amount of TSL and GTSL solution. Then the surface binding of gold nanoclusters and phospholipids was analyzed by energy dispersive X-ray spectroscopy (EDS).

#### Ultraviolet spectrum scanning

The GTSL and GTSL-HER2 solutions were diluted to to a concentration of 1 mg/mL. And the solution was scanned with an ultraviolet–visible spectrophotometer in the range of 400-1000 nm.

### The temperature change after laser irradiation

#### Continuous laser irradiation

GTSL, GTSL-CYC, GTSL-HER2, GTSL-CYC-HER2 were diluted to 10 mg/ml and was irradiated (3 W/cm^2^) for 10 min, PBS and TSL as controls. After 10 min of continuous laser irradiation with a power of 3 W/cm^2^, an infrared thermal imager was recorded to monitor the temperature change in real time, and to investigate photothermal converting ability under NIR irradiation. In addition,

#### Pulsed laser irradiation

The solutions of GTSL, GTSL-CYC, GTSL-HER2 and GTSL-CYC-HER2 were diluted to 10 mg/mL and irradiated with NIR irradiation (3 W/cm^2^) respectively. The temperature of the solution was recorded by continuous irradiation for 10 min. After the temperature returned to the initial temperature, the above circular irradiation was repeated for five times. The the temperature changes in each irradiation period were recorded with an infrared thermal imaging instrument to investigate the photothermal effect of gold nanoclusters on the surface of TSL.

#### Calculation of photothermal conversion efficiency

The preparations were diluted to 1 mg/ml and placed in a colorimetric dish to weigh the mass of the sample (m). The samples above were measured at 808 nm for their ultraviolet absorbance value (A), and then were irradiated with an 808 nm laser (3 W/cm^2^) for 540 s, followed with natural cooling time of 540 s. The temperature changes were recorded by an infrared thermal imager every 30 s, purified water as a blank control. The photothermal conversion efficiency (η) is calculated by the following formula [30]:

$$ \theta = \frac{{T - T_{surr} }}{{T_{max} - T_{surr} }} $$

$$ {{\text{k}}} = \frac{ - \ln \theta }{{\text{t}}} $$

$$ hs = \frac{{mc_{water} }}{k} $$

$$ {\text{Q}}_{{{\text{dis}}}} = \frac{{{\text{mc}}_{{{\text{water}}}} \left( {{\text{T}}_{{\max \left( {{\text{water}}} \right)}} - {\text{T}}_{{{\text{surr}}}} } \right)}}{{{\uptau }_{{{\text{water}}}} }} $$

$$ {\upeta } = \frac{{hs\left( {T_{max} - T_{surr} } \right) - Q_{dis} }}{{I\left( {1 - 10^{ - A} } \right)}} $$

where H is the heat transfer coefficient, S is the surface area of the container, T_max_ is the highest temperature at which the sample solution is heated up, T_surr_ is the ambient temperature at which the sample is tested, I is the power density of the laser, and A is the absorption intensity of the sample at 808 nm. M is the mass of the sample solution, and C_water_ is the specific heat capacity of water. In order to calculate hs, θ is introduced in this experiment to define k, that k is the slope of the line (t- -lnθ). In the formula, Q_dis_ is the value of blank sample, and T_max_(water) is the maximum temperature of water after the illumination. K_water_ represented the slope between -lnθ of water and irradiation time.

#### The analysis of release behavior

The PBS solution containing 20% PEG was prepared as release medium and was adjusted to pH 7.4 and pH 5.4 to simulate normal blood environment and lysosome environment. 0.8 ml of CYC, TSL-CYC, GTSL-CYC, TSL-CYC@NIR, GTSL-CYC@NIR, TSL-CYC-HER2, GTSL-CYC-HER2, TSL-CYC-HER2@NIR and GTSL-CYC-HER2@NIR solution were placed in the dialysis bag, respectively. Then the dialysis bags were immersed in a centrifuge tube containing 8 mL of release medium and shake with the speed of 100 rpm at 37℃. Among that, the laser irradiation group TSL-CYC@NIR, GTSL-CYC@NIR, TSL-CYC-HER2@NIR and GTSL-CYC-HER2@NIR were taken out of the centrifuge tube at specific points (0, 2, 4, 6 h), to give a continuous irradiation for 5 min, and then put it back into the centrifuge tube. All the above samples were taken 0.1 ml of releasing medium for further detection and replace it with fresh medium at 0.5, 1, 2, 4, 6, 8, 12, 24, 36, 48 h, respectively. The CYC in the samples was detected by HPLC, which was performed on RP-18 column (Zorbax Eclipse Plus, 5 μm, 250 mm × 4.6 mm, Agiligent), mobile phase was methanol-ammonium acetate (0.05 M) (70:30); and the detection wavelength was set at 210 nm, the flow rate was 1 Ml/min, the column temperature was set at 25℃, and the sample size was 20 μL, after determination the cumulative release curve was plotted.

### The degradation behavior of GTSL-CYC-HER2

#### The degradation of GTSL-CYC-HER2 after laser irradiation

The GTSL-CYC-HER2 was irradiated with the NIR laser with a power of 3.0 W/cm^2^. After continuous irradiation for 10 min, the morphology and particle size of GTSL-CYC-HER2 before and after the irradiation were observed with a transmission electron microscope for comparison。

#### Degradation of GTSL-CYC-HER2 in simulated body fluids and simulated lysosomal environments

Na_2_HPO_4_ 142.0 mg, NaCl 6.65 g, Na_2_SO_4_ 71 mg, CaCl_2_.2H_2_O 29 mg, glycine 450 mg, potassium hydrogen phthalate 4.084 g and benzalkonium chloride 50 mg were dissolved in 1L water as SLF solution to stimulate lysosome environment. Each 1 mL of GTSL-CYC-HER2 was placed in 10 mL of simulated body fluid (SBF, pH 7.4) and SLF (pH 4.0), respectively. After co-incubated in a shaker of 180 rpm at 42℃ for 48 h, the morphology and particle size of GTSL-CYC-HER2 were observed by TEM at 0, 24 and 48 h respectively.

### The evaluation on cell level

#### The expression of HER2 on various breast tumor cells

In this study, the expression of HER2 were determined by Western blot method and Immunofluorescence method in MCF-7、MDA-MB-453、BT474 and SK-BR-3 cells. Based on the expression content, low-expressing and high-expressing cells were screened for further study.

#### The cytotoxicity assay in MCF-7 and SK-BR-3 cells

The cells were seeded in a 96-well plate at a density of 10,000 per well. After 24 h incubation, the following preparations diluted in different concentrations were added respectively, including TSL-CYC, GTSL-CYC, GTSL-CYC-HER2, TSL@NIR, GTSL@NIR, GTSL-HER2@NIR, TSL-CYC@NIR、GTSL-CYC@NIR、GTSL-CYC-HER2@NIR, the zero hole and PBS as blank control groups. Among that, TSL@NIR, GTSL@NIR, GTSL-HER2@NIR, TSL-CYC@NIR, GTSL-CYC@NIR, GTSL-CYC-HER2@NIR was firstly placed in the incubation for 4 h, and then was irradiated for 5 min with 808 nm laser (3.0 W/cm^2^). After the irradiation, put it in the incubation and continue culture, similar with other group. With other 20 h incubation, 10 μL of 0.5% MTT solution was added to each well and continue culturing for 4 h. Finally, the solution in the holes was removed and washed with PBS for three times, followed with addition of 150μL of DMSO to solve the crystal. The absorbance of each well was determined by enzyme-labeled instrument at the OD 570 nm. The cell viability could be calculated according to formula

$$ {\text{Cell viability \% }} = \frac{{A_{sample} - A_{blank} }}{{\left( {A_{control} - A_{blank} } \right)}} \times 100\% $$

where, A_sample_ represent sbsorbance value of the sample while A_blank_ represented the sbsorbance value of zero hole. A_control_ is the absorbance value of the control hole。

#### Study on synergistic index of chemotherapy combined with hyperthermia

The synergistic effects of chemotherapy and hyperthermia were studied via Compusyn software. According to the report of Chou and Talalay, the Combination Index (CI) was used as the Index to evaluate the synergistic effect[31]. As shown in Table 2, this standard was used to determine whether GTSL-CYC@NIR had synergistic effects in MCF-7 cells and SK-BR-3 cells.

**Table 2:** synergism in combination studies analyzed with the combination index method

Range of combination index	Description
< 0.1	Very strong synergism
0.1–0.3	Strong synergism
0.3–0.7	Synergism
0.7–0.85	Moderate synergism
0.85–0.90	Slight synergism
0.90–1.10	Nearly additive
1.10–1.20	Slight antagonism
1.20–1.45	Moderate antagonism
1.45–3.3	Antagonism

#### Cell uptake study of GTSL-CYC-HER2 on tumor cells

The TSL-C6 and TSL-C6-HER2 loading fluorescein C6 were prepared by the thin film dispersion method. The MCF-7 cells and SK-BR-3 cells were seeded in 6-well plates. After 24 h incubation, the medium was removed and the free C6, TSL-C6 and TSL-C6-HER2 prepared were added with the concentration of C6 of 100 ng/ml. At 2, 4, and 6 h, the holes were washed three timse with PBS, and were placed under an inverted fluorescence microscope to observe the uptake content. In addition, the above experiment was repeated. At 2, 4, and 6 h, the cells in hole were all collected and re-suspended in 200μL of PBS to determine the fluorescence content with a flow cytometer.

To evaluate the uptake of gold clusters in MCF-7 cells and SK-BR-3 cells, the uptake content of Au was determined by ICP-MS. Firstly, the MCF-7 cells and SK-BR-3 cells in the logarithmic growth phase were seeded in 24-well culture plate at 1.25 × 10^5^ cells/well. After 24 h, the GTSL-CYC, GTSL-CYC-HER2 and GTSL-CYC-HER2@NIR irradiation groups (3 W/cm2, 5 min/well) were added and incubated. At 2, 4, and 6 h, the cells in holes were collected and lysed by 60 μL RIPA (containing 1 mM PMSF). After complete lysis, the protein in the lysates was examined by BCA kit. Moreover, the Au content is determined by ICP-MS, and the Au content per unit protein is calculated to investigate the uptake behavior.

#### Uptake inhibition experiment after HER2 receptor saturation

To investigate the uptake mechanism of GTSL-CYC-HER2, the receptor inhibition experiment was carried out. The SK-BR-3 cells in the logarithmic growth phase were seeded in 24-well culture plate at 1.25 × 10^5^ cells/well. The GTSL-CYC-HER2@NIR samples(3 W/cm2, 5 min/well) were added. In addition, the control group was firstly added with excess of HER2 antibody, and then the GTSL-CYC-HER2@NIR samples (3 W/cm2, 5 min/well) were added. At 2 h, 4 h, and 6 h, the cells in holes were collected and lysed by 60 μL RIPA (containing 1 mM PMSF). After complete lysis, the protein in the lysates was examined by BCA kit. Moreover, the Au content is determined by ICP-MS, and the Au content per unit protein is calculated to investigate the uptake behavior.

#### The penetration behavior in the tumor-sphere model

An appropriate amount of 1 mg/mL SH-PEG-FITC solution was mixed with GTSL at mass ratio of 1:1. The mixture was stirred overnight in the dark and the FITC was labelled via Au–S bond on the surface of the nanoclusters, and then purified by ultrafiltration through ultrafiltration centrifuge tube (10 kDa, Millipore) for three times to remove unconjugated FITC. Then GTSL-F was obtained for further use.

50 μl of tumor cells culture medium (SK-BR-3) were dropped onto the petri dishes, and then placed upside down in a cell incubator at 37℃ and 5% CO_2_. After 24 h, the inverted tumor globule was transferred to the agarose culture concave layer, and 150ul of complete culture was added to continue to culture tumor globules. Then, the tumor globules were divided into 3 groups and added the free SH-PEG-FITC, GTSL-F and GTSL-F@NIR respectively. After 4 h incubation, the NIR group was irradiated with near-infrared laser at 3 W/cm2 for 5 min and continued to incubate for 12 h. After 12 h, the culture medium was removed and the tumor globules were washed for 3 times with cold PBS solution. Next, the tumor globules were fixed with 4% paraformaldehyde for 30 min, and rinsed with PBS for 3 times. Finally, the tumor spheres were moved to the confocal plate, and z-STACK tomography was performed with laser confocal microscopy to observe the penetration of free SH-PEG-FITC, GTSL-F and GTSL-F@NIR.

### In vivo study

#### Establishment of in-situ breast cancer-bearing mice model

The SK-BR-3 cells in the logarithmic growth phase were collected and suspended in PBS at the density of 1 × 10^7^/mL cell. Then 0.1 ml of the solution was injected into the fourth pair of mammary fat pads of Balb/c nude mice subcutaneously, to establish the in situ breast cancer model. All animal experiments were conducted in accordance with the guidelines of the Animal Ethics Committee of China Pharmaceutical University.

#### The tumor targeting ability in vivo

The breast cancer mice were randomly divided into three groups, 4 mice in each group, and were injected intravenously with Free-Dir, Dir@GTSL, and Dir@GTSL-HER2 at a dose of 1 mg/kg (CYC). Next, the images were recorded by In vivo imaging (Dir, excitation wavelength 745 nm, emission wavelength 800 nm) at 2, 8, and 24 after administration. At 24 h, the nude mice were sacrificed, and the heart, liver, spleen, lung, kidney, brain and tumor were obtained and photographed by In vivo imaging to calculate the fluorescence intensity value, observing the targeting ability of the samples.

### The photothermal properties in vivo

The breast cancer mice were randomly divided into three groups, 4 mice in each group, and were injected intravenously with saline, GTSL and GTSL-HER2 at a dose of 10 mg/kg (Au) by tail vein. After 24 h, the tumor tissue was irradiated with a 808 nm laser (3 W/cm2) for 5 min. An infrared thermal imager was used to monitor the temperature change of the tumor in real time.

### CT imaging studies in vivo

The breast cancer mice were randomly divided into three groups, 4 mice in each group, and were injected intravenously with 0.2 ml iohexol, GTSL and GTSL-HER2 at a dose of 10 mg/kg (Au) by tail vein. The scans were performed on PET/CT before injection and 2 and 8 h after injection. The instrument parameters were set as follows: tube voltage 80 kV, current intensity 500 μA, and single exposure time 250 ms. The tumor-bearing nude mice were sacrificed immediately after CT scan. After dissection, the tumor and main organ tissues (heart, liver, spleen, lung, kidney, brain, tumor) were obtained and weighed. Then the organs were digested using microwave digestion equipment to determine the gold content by ICP-MS. Finally, the percent cumulative amount of different preparations in different tissues %ID/g (percentage of injected dose/g) was calculated.

#### The antitumor study in vivo

The breast cancer mice were randomly divided into seven groups, 6 mice in each group, and were injected intravenously with the following samples, including normal saline group, light group (NIR group), free drug group (CYC), GTSL@NIR group, GTSLs-CYC@NIR group, GTSL-CYC-HER2 group, GTSL-CYC-HER2@NIR group. The mice of each group was administered by tail vein injection at a dose of 10 mg/kg (at the base of CYC), and the amount of tail vein injection was 0.1 mL/10 g. The drug was firstly administered from the second week and then every 3 days for a total of 4 doses. The laser power of the laser irradiation group was set at 3 W/cm^2^ for 5 min after the administration. The tumor volume and weight of mice were monitored every 3 day to observe the antitumor effect. On the 14th day, the Balb/c nude mice were sacrificed. The tumor tissues were dissected and immersed in 4% paraformaldehyde solution, dehydrated, embedded, and sliced for further Hematoxylin–Eosin (H&E) Staining and TUNEL staining.

#### The safety study in *vivo*

The breast cancer mice were randomly divided into seven groups, 6 mice in each group, and were injected intravenously with the following samples, including normal saline group, light group (NIR group), free drug group (CYC), GTSL@NIR group, GTSL-CYC@NIR group, GTSL-CYC-HER2 group, GTSL-CYC-HER2@NIR group. The mice of each group was administered by tail vein injection at a dose of 10 mg/kg (at the base of CYC), and the amount of tail vein injection was 0.1 mL/10 g. The drug was firstly administered from the second week and then every 3 days for a total of 4 doses. On the 14th day, the Balb/c nude mice were sacrificed. In order to evaluate the damage to the tissue of the therapy, all the organs were obtained and immersed in 4% paraformaldehyde solution, dehydrated, embedded, and sliced for further Hematoxylin–Eosin (H&E) Staining.

## Supplementary Information

**Additional file 1**:** Figure S1.** Molecular weight of HER2 by SDS-PAGE electrophoresis.** Figure S2.** Images of GTSL prepared by the reduction of HAuCl4 solution with ascorbic acid solution and the volume of HAuCl4 added from left to right is 18 μL(a), 24 μL(b), 60 μL(c).** Figure S3.** Photothermal properties of the GTSL (A), GTSL-CYC-HER2 (C) solutions at 808nm (3W/cm^2^), then the irradiation lasted for 540s and was then shut off. Plot of the cooling time vs -lnθ from the cooling stage of GTSL (B), GTSL-CYC-HER2 (D).** Figure S4.** Western blot analysis of HER-2 protein among four different breast cancer cells (**p<0.01).** Figure S5.** Post-light toxicity test of different cells.** Figure S6.** Dose-Effect Curve of MCF-7 cells (A); Combination Index Plot (B).** Figure S7.** Dose-Effect Curve of SK-BR-3 cells (A); Combination Index Plot (B).** Figure S8.** Quantitative comparison of GTSL-CYC, GTSL-CYC-HER2 and GTSL-CYC-HER2@NIR uptaken into MCF-7(A) and SK-BR-3 cells (B) at 2, 4, 6h by ICP-MS; C. The content of GTSL-CYC-HER2 @NIR uptake into MCF-7 and SK-BR-3 cells was compared at 2, 4, and 6 h (C). (**p < 0.01, ***p < 0.001).** Figure S9.** The penetration behavior of different preparations into the tumor sphere.** Figure S10.** The preliminary pharmacokinetic behavior of agents in vivo.** Figure S11.** The images of ex vivo organ of mice at 96 h after injection of GTSL and GTSL-HER2.** Figure S12.** The weight changes of mice in all at 14 days.** Figure S13.** The Hematoxylin and eosin (H&E) staining of organs after treatment.** Table S1.** Effect of different GN content on the UV maximum absorption wavelength of GTSL.** Table S2.** Photothermal conversion parameters measurement results.** Table S3.** Gray values of HER2 protein expression among four different cancer cells.** Table S4.** MCF-7 CI values for actual experimental points.** Table S5.** SK-BR-3 CI values for actual experimental points.
