# Supplementary material for: “Petal-like” size-tunable gold wrapped immunoliposome to enhance tumor deep penetration for multimodal guided two-step strategy
Source: J Nanobiotechnology. 2021 Sep 27;19:293. doi: 10.1186/s12951-021-01004-1 (PMC8477504; doi:10.1186/s12951-021-01004-1)
Supplement: Supplementary file 1 — Additional file 1: Figure S1. Molecular weight of HER2 by SDS-PAGE electrophoresis. Figure S2. Images of GTSL prepared by the reduction of HAuCl4 solution with ascorbic acid solution and the volume of HAuCl4 added from left to right is 18 μL(a), 24 μL(b), 60 μL(c). Figure S3. Photothermal properties of the GTSL (A), GTSL-CYC-HER2 (C) solutions at 808nm (3W/cm2), then the irradiation lasted for 540s and was then shut off. Plot of the cooling time vs -lnθ from the cooling stage of GTSL (B), GTSL-CYC-HER2 (D). Figure S4. Western blot analysis of HER-2 protein among four different breast cancer cells (**p<0.01). Figure S5. Post-light toxicity test of different cells. Figure S6. Dose-Effect Curve of MCF-7 cells (A); Combination Index Plot (B). Figure S7. Dose-Effect Curve of SK-BR-3 cells (A); Combination Index Plot (B). Figure S8. Quantitative comparison of GTSL-CYC, GTSL-CYC-HER2 and GTSL-CYC-HER2@NIR uptaken into MCF-7(A) and SK-BR-3 cells (B) at 2, 4, 6h by ICP-MS; C. The content of GTSL-CYC-HER2 @NIR uptake into MCF-7 and SK-BR-3 cells was compared at 2, 4, and 6 h (C). (**p < 0.01, ***p < 0.001). Figure S9. The penetration behavior of different preparations into the tumor sphere. Figure S10. The preliminary pharmacokinetic behavior of agents in vivo. Figure S11. The images of ex vivo organ of mice at 96 h after injection of GTSL and GTSL-HER2. Figure S12. The weight changes of mice in all at 14 days. Figure S13. The Hematoxylin and eosin (H&E) staining of organs after treatment. Table S1. Effect of different GN content on the UV maximum absorption wavelength of GTSL. Table S2. Photothermal conversion parameters measurement results. Table S3. Gray values of HER2 protein expression among four different cancer cells. Table S4. MCF-7 CI values for actual experimental points. Table S5. SK-BR-3 CI values for actual experimental points. [file 12951_2021_1004_MOESM1_ESM.docx]

**Supporting Information**

**Size-tunable gold wrapped immunoliposome to enhance tumor deep penetration for two-step strategy**

Yanan Li^a,b#^, Wenting Song ^a#^, Yumin Hu^a#^, Yun Xia^a^, Zhen L^ia^, Yang Lu^c*^, Yan Shen^a*^

aDepartment of Pharmaceutics, School of Pharmacy, China Pharmaceutical University, 210009, P. R. China

bSchool of Food Science and Pharmaceutical Engineering, Nanjing Normal University, Nanjing, 210023, P. R. China

cLaboratory of Traditional Chinese Medicine, School of Chinese Materia Medica, Beijing University of Chinese Medicine, Beijing, 100029，China

#These authors contributed equally to this work.

*Corresponding author.

E-mail addresses: Yan Shen, [shenyan@cpu.edu.cn](mailto:shenyan@cpu.edu.cn); Yang Lu, landocean28@163.com

**Figure S1**


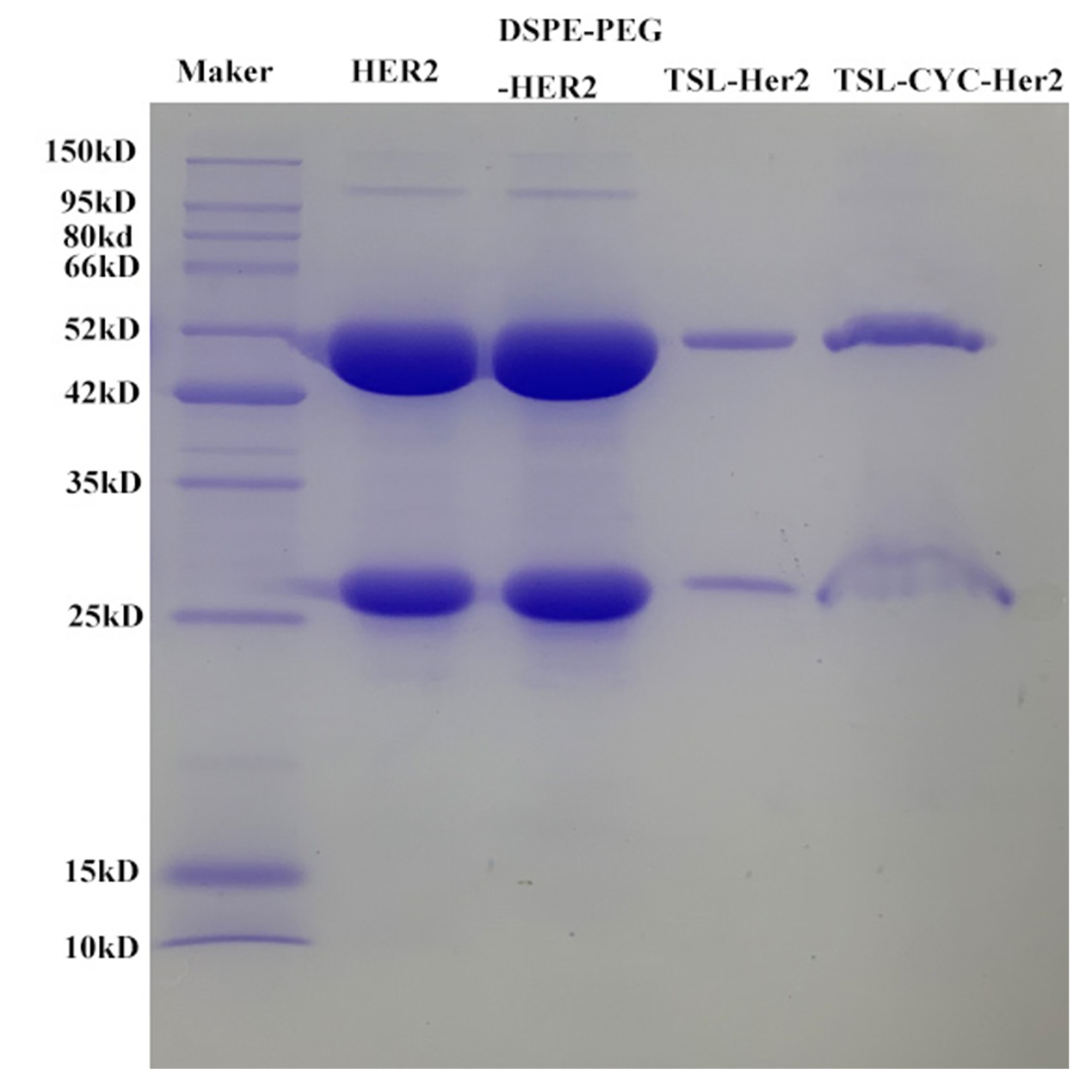


Figure S1. Molecular weight of HER2 by SDS-PAGE electrophoresis.

**Figure S2**


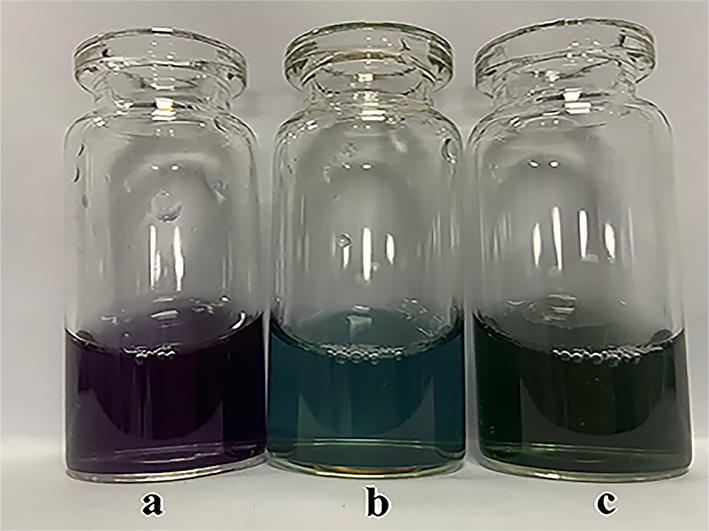


Figure S2. Images of GTSL prepared by the reduction of HAuCl_4_ solution with ascorbic acid solution and the volume of HAuCl_4_ added from left to right is 18 μL(a), 24 μL(b), 60 μL(c).

**Figure S3**


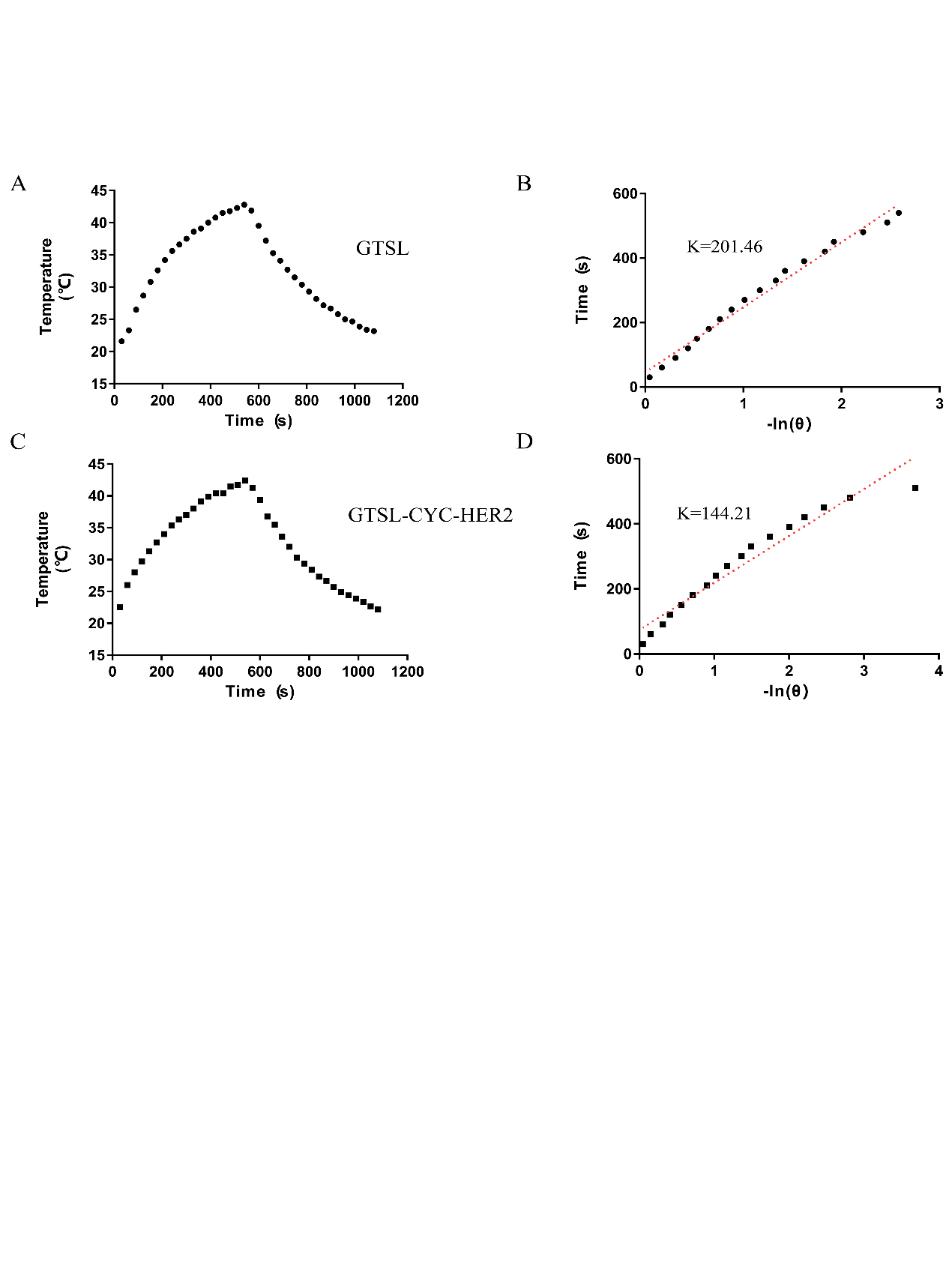


Figure S3.Photothermal properties of the GTSL (A), GTSL-CYC-HER2 (C) solutions at 808nm (3W/cm^2^), then the irradiation lasted for 540s and was then shut off. Plot of the cooling time vs -lnθ from the cooling stage of GTSL (B), GTSL-CYC-HER2 (D).

**Figure S4**

**
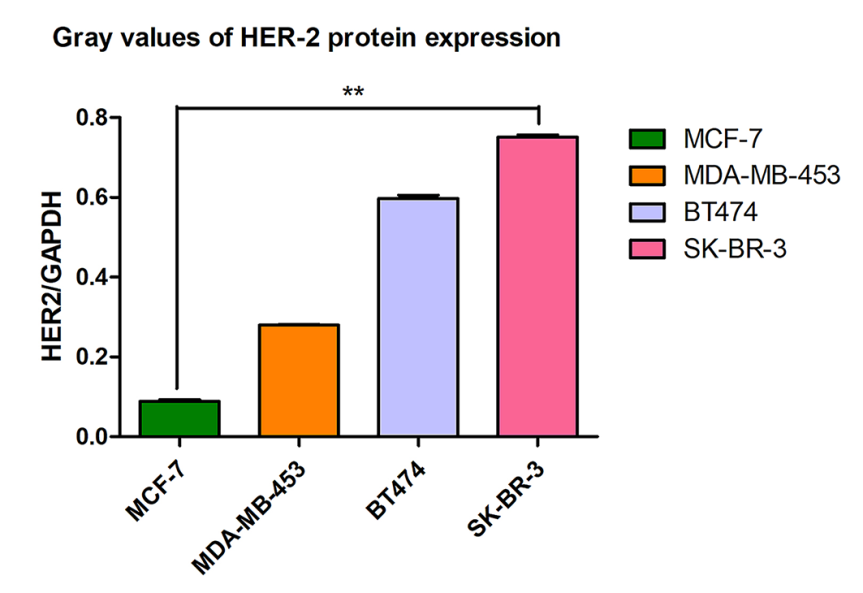
**

Figure S4 Western blot analysis of HER-2 protein among four different breast cancer cells(**p<0.01).

Figure S5


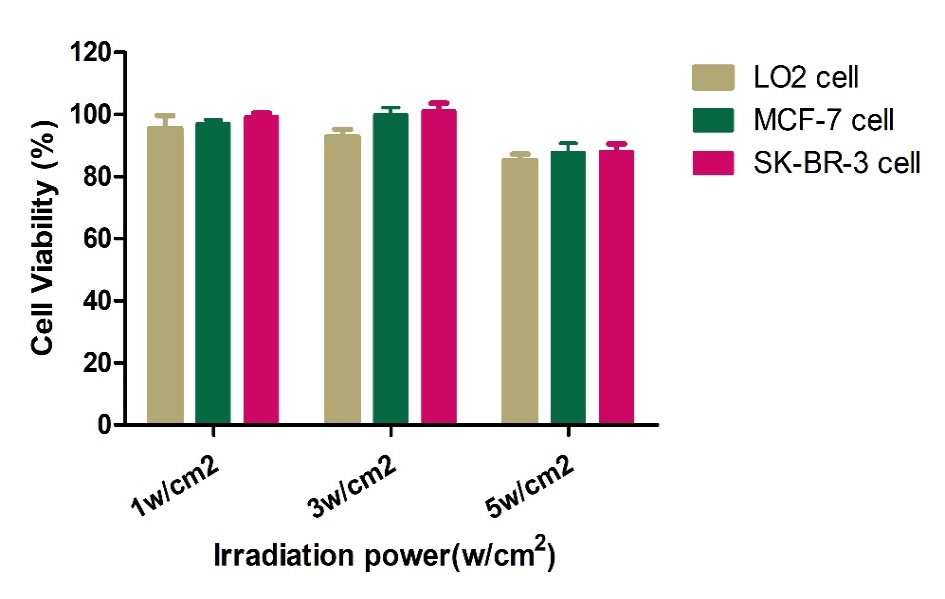


Figure S5. Post-light toxicity test of different cells.

**Figure S6**


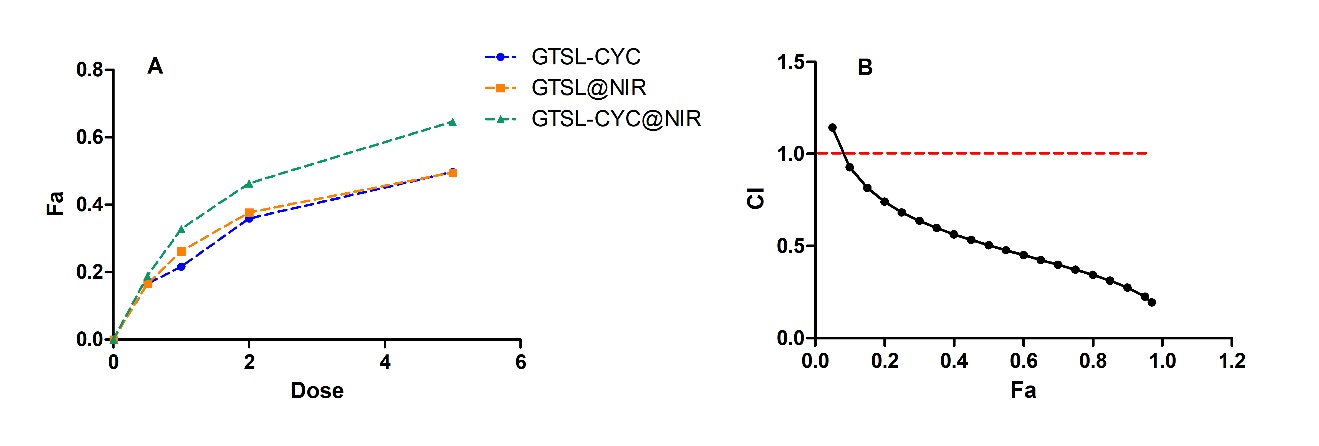


Figure S6. Dose-Effect Curve of MCF-7 cells (A); Combination Index Plot (B).

**Figure S7**


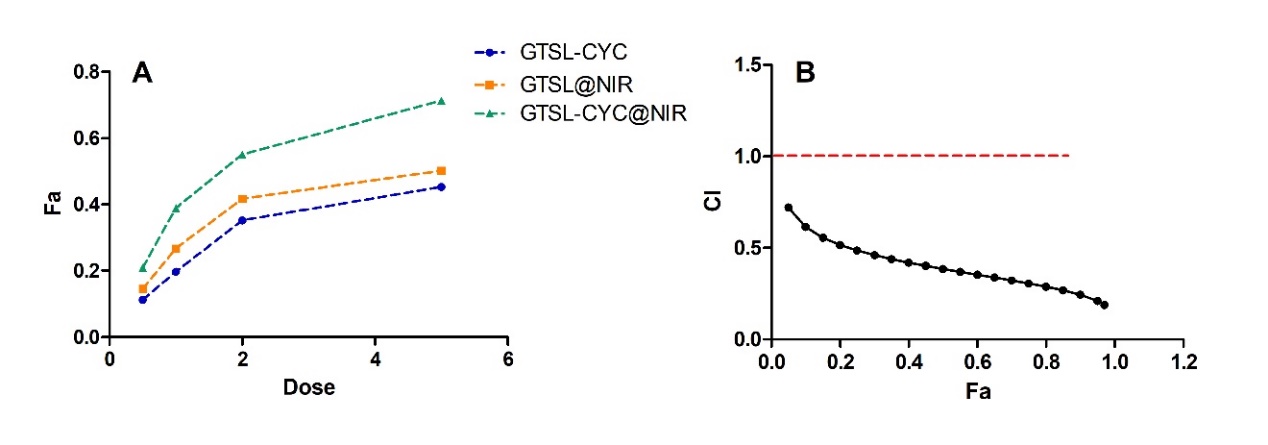


Figure S7. Dose-Effect Curve of SK-BR-3 cells (A); Combination Index Plot (B).

**Figure S8**


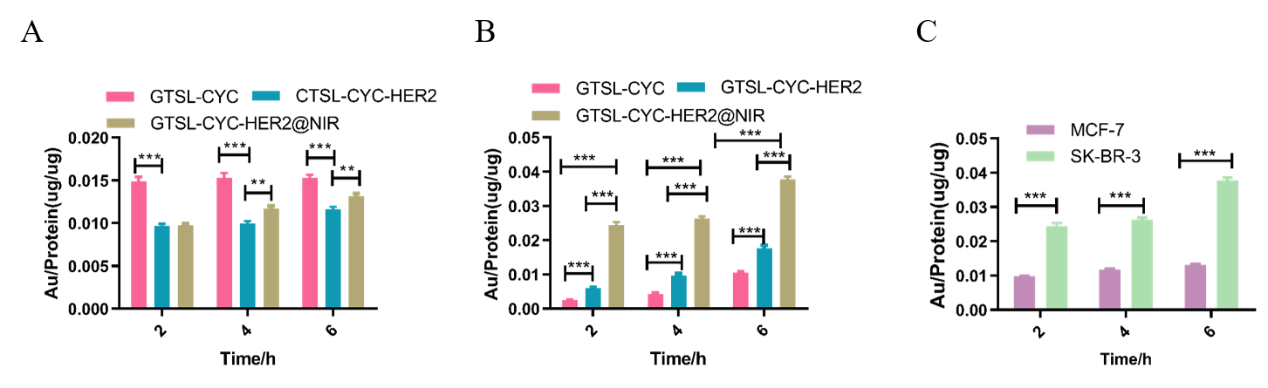


Figure S8. Quantitative comparation of GTSL-CYC, GTSL-CYC-HER2 and GTSL-CYC-HER2@NIR uptaken into MCF-7(A) and SK-BR-3 cells (B) at 2, 4, 6h by ICP-MS ; C. The content of GTSL-CYC-HER2 @NIR uptake into MCF-7 and SK-BR-3 cells was compared at 2, 4, and 6 h (C). (**p＜0.01，***p＜0.001).

**Figure S9**


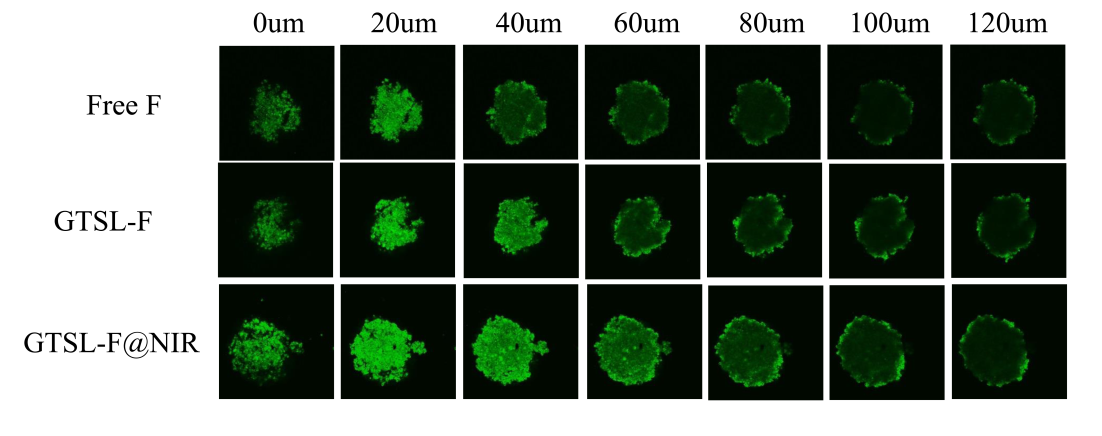


**Figure S9** The penetration behavior of different preparations into the tumor sphere.

**Figure S10**


**Figure S10** The preliminary pharmacokinetic behavior of agents in vivo.

**Figure S11**


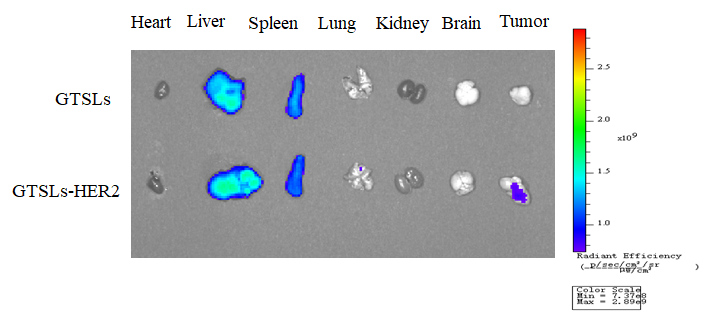


Figure S11 The images of *ex vivo* organ of mice at 96 h after injection of GTSL and GTSL-HER2.

**Figure 12**


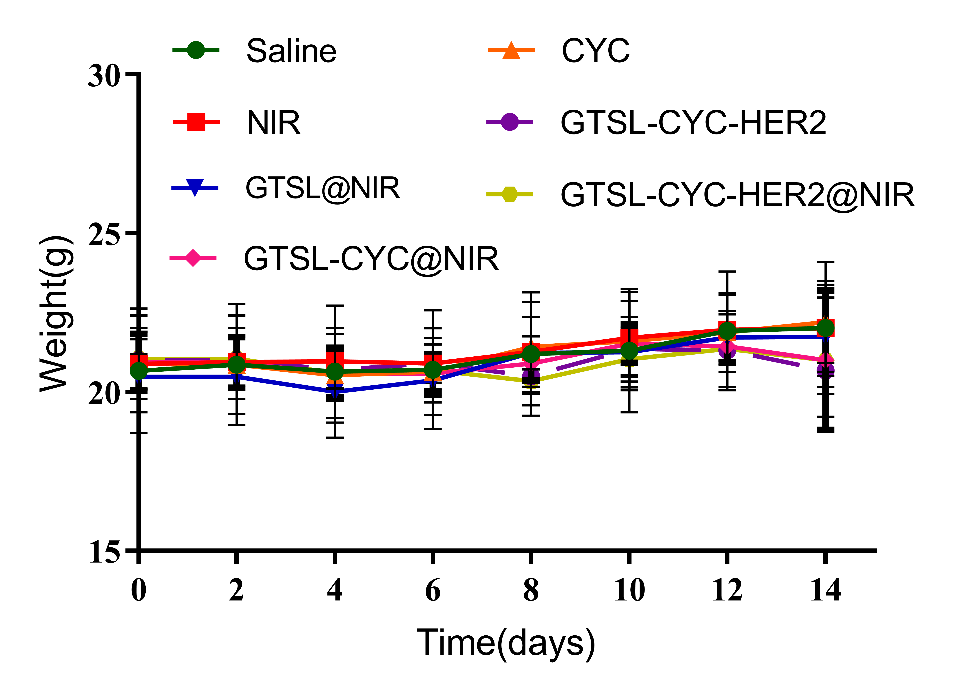


Figure S12. The weight changes of mice in all at 14 days.

**Figure S13**


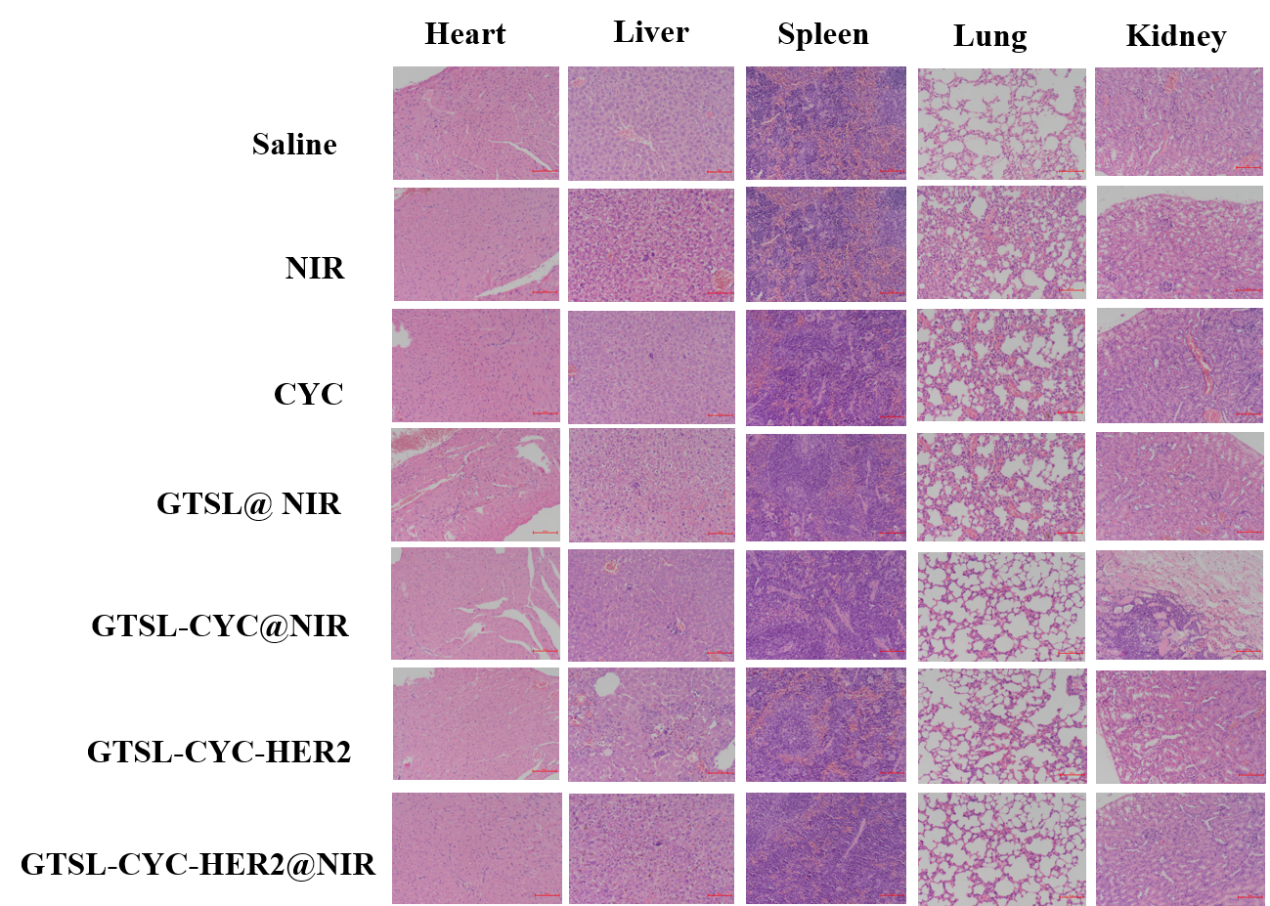


Figure S13. The Hematoxylin and eosin (H&E) staining of organs after treatment.

**Table S1**

Table S1 Effect of different GN content on the UV maximum absorption wavelength of GTSL

| HAuCl_4_  (μL) | Ascorbic Acid  (μL) | UV (nm) | Diameters (nm) |
| --- | --- | --- | --- |
| 16 | 24 | 657 | 174.9 |
| 20 | 30 | 735 | 159.1 |
| **24** | **36** | **792** | **113.5** |
| 30 | 45 | 862 | 138.9 |
| 40 | 60 | 1023 | 237.2 |
| 50 | 75 | >1100 | 424.5 |

**Table S2**

Table S2 Photothermal conversion parameters measurement results

| Sample | k | m(g) | T_max_ (℃) | T_surr_ (℃) | Q_dis_ (mw) | Abs.(nm) | η (%) |
| --- | --- | --- | --- | --- | --- | --- | --- |
| GTSL | 201.46 | 1.712 | 42.8 | 21.6 | 8.31 | 0.593 | 30.14 |
| GTSL-CYC-HER2 | 144.21 | 1.666 | 42.4 | 22.2 | 8.09 | 0.677 | 37.96 |

**Table S3**

Table S3 Gray values of HER2 protein expression among four different cancer cells

|  | MCF-7 | MDA-MB-453 | BT474 | SK-BR-3 |
| --- | --- | --- | --- | --- |
| HER2 | 987.0 | 2735.5 | 5659.0 | 7209.5 |
| GAPDH | 10321 | 9666.4 | 9497.1 | 9711.2 |
| HER2/GAPDH | 0.095 | 0.283 | 0.596 | 0.742 |

**Table S4**

Table S4 MCF-7 CI values for actual experimental points

| Total Dose | Fa | CI Value |
| --- | --- | --- |
| 0.5 | 0.189 | 0.806 |
| 1.0 | 0.328 | 0.569 |
| 2.0 | 0.464 | 0.508 |
| 5.0 | 0.646 | 0.445 |

**Table S5**

Table S5 SK-BR-3 CI values for actual experimental points

| Total Dose | Fa | CI Value |
| --- | --- | --- |
| 0.5 | 0.209 | 0.571 |
| 1.0 | 0.328 | 0.380 |
| 2.0 | 0.550 | 0.337 |
| 5.0 | 0.713 | 0.347 |
